# Empagliflozin restores cardiac function in obese-diabetic HFpEF mice but further alters gene expression

**DOI:** 10.1007/s00395-026-01184-7

**Published:** 2026-05-21

**Authors:** Juliana Mira Hernandez, Logan R. J. Bailey, Victoria R. Salemme, Christopher Y. Ko, Erin Y. Shen, Megan Ngim, Duong T. Hoang, Melissa Corea, Julie Bossuyt, Jennifer M. Davis, Donald M. Bers, Bence Hegyi

**Affiliations:** 1 Department of Pharmacology, University of California, Davis, 451 Health Sciences Drive, Davis, CA 95616 USA; 2 Research Group Biogenesis, Faculty of Agricultural Sciences, Veterinary Medicine, University of Antioquia, Medellin, Colombia; 3 Institute for Stem Cell and Regenerative Medicine, University of Washington, Seattle, WA USA; 4 Department of Lab Medicine and Pathology, University of Washington, Seattle, WA USA

**Keywords:** Preclinical animal model, HFpEF, SGLT2 inhibitor, Ion channels, Excitation–contraction coupling, Gene transcription

## Abstract

Effective treatments for heart failure with preserved ejection fraction (HFpEF) are limited. Empagliflozin, a sodium-glucose cotransporter-2 (SGLT2) inhibitor, provides cardiovascular benefits for patients with HFpEF. SGLT2 expression is virtually absent in cardiomyocytes, hence the exact protective mechanism of empagliflozin is unclear. We aimed to study the effect of in vivo empagliflozin treatment on excitation–contraction coupling (ECC), electrophysiology, and gene transcription in a translational two-hit HFpEF mouse model that combines the obese-diabetic leptin receptor-deficient *db/db* mice with chronic aldosterone infusion (*db/db* + Aldo). We performed morphometry, echocardiography, cardiomyocyte electrophysiology, intracellular Ca^2+^ imaging, and whole-heart RNA-sequencing in empagliflozin-treated *db/db* + Aldo and vehicle control mice. In *db/db* + Aldo mice, empagliflozin (in vivo, 4 weeks) reduced the elevated plasma blood glucose (13 ± 5 mmol/L *versus* 23 ± 8 mmol/L) and BNP levels, prevented pulmonary congestion, and ameliorated diastolic dysfunction. However, empagliflozin did not reduce cardiac hypertrophy and obesity. In healthy control mice, empagliflozin had no functional effect. Chronic in vivo empagliflozin treatment in *db/db* + Aldo also normalized the changes in cardiomyocyte electrophysiology and Ca^2+^ handling properties measured in the absence of empagliflozin in the perfusion solution during cell experiments, indicating a true phenotypic rescue. Differentially expressed gene clusters were identified characteristic of the *db/db* genotype, aldosterone, and empagliflozin treatments. Interestingly, empagliflozin did not just reverse the HFpEF-altered gene expression but shifted it further away from control to a new expression profile. We conclude that chronic in vivo empagliflozin treatment restores cardiomyocyte electrophysiological and ECC properties, ameliorates diastolic function in cardiometabolic HFpEF, and induces complex gene expression changes.

**Supplementary Information:**

The online version contains supplementary material available at 10.1007/s00395-026-01184-7.

## Introduction

Heart failure (HF) with preserved ejection (HFpEF) accounts for ≥ 50% of all HF cases, has high mortality rates, and has limited effective therapeutics available [6]. Empagliflozin, a sodium-glucose cotransporter-2 (SGLT2) inhibitor, was the first drug that showed benefits in patients with HFpEF in clinical trials [1]. Empagliflozin is a potent antidiabetic medication; however, clinical and experimental evidence demonstrates that empagliflozin provides cardiovascular benefits beyond glycemic control [12]. Importantly, SGLT2 expression is virtually absent in cardiomyocytes, yet empagliflozin has multiple beneficial effects in ex vivo perfused hearts and in isolated myocytes, indicating off-target effects [15, 35]. In fact, empagliflozin has shown protective effects against developing HF with reduced ejection fraction (HFrEF) even in SGLT2 knockout mice [4, 9]. Empagliflozin improves diastolic function and may reduce arrhythmias in patients with type 2 diabetes mellitus (T2DM) with or without HFpEF and in animal models [8, 44, 48, 64]; however, the exact molecular mechanisms remain incompletely understood.

Excitation–contraction coupling (ECC) impairments and ion channel remodeling play key roles in HF, contributing to contractile dysfunction and arrhythmia [47]. Empagliflozin has been implicated in regulating ionic movements and ECC in cardiomyocytes [18], including cardiac Na^+^/H^+^ exchanger (NHE-1) [2, 9], late Na^+^ current (I_Na,L_) [24, 46], and Ca^2+^/calmodulin-dependent protein kinase II (CaMKII) [38, 39] as putative targets of empagliflozin. However, there are still controversies about these being direct targets of empagliflozin [10, 24, 37, 65]. Empagliflozin pretreatment but not acute application attenuated the enhanced I_Na,L_ and CaMKII activity in failing myocytes [24, 39]. Empagliflozin also reduced the level of reactive oxygen species (ROS) [32] and intracellular *O*-linked β-N-acetylglucosamine (*O*-GlcNAc) modified proteins [29]. Both ROS and *O*-GlcNAc are important post-translational regulatory mechanisms of ECC, ion channels, and CaMKII that have been implicated in diabetes and HF [19].

We aimed to determine the chronic in vivo effects of empagliflozin on cardiomyocyte electrophysiology, ECC, and transcriptome profile in a two-hit cardiometabolic HFpEF mouse model that combines the obese-diabetic leptin receptor-deficient *db/db* mice with chronic aldosterone (Aldo) infusion (*db/db* + Aldo) [23, 36]. We and others have previously shown that empagliflozin treatment of isolated HFpEF cardiomyocytes reversed proarrhythmogenic action potential (AP) changes and I_Na,L_ enhancement [23, 24, 36, 38]; however, it was unclear whether empagliflozin provides functional ECC benefits and whether it could reverse cardiac remodeling in HFpEF. We hypothesized that in vivo empagliflozin treatment in HFpEF will restore cardiomyocyte electrophysiology and ECC mechanisms and attenuate proarrhythmia even when cardiomyocytes are bathed in the absence of empagliflozin, indicating a lasting phenotypic rescue.

## Methods

Animal handling and laboratory procedures followed the approved protocol of the Institutional Animal Care and Use Committee at the University of California, Davis (#23175) conforming to the Guide for the Care and Use of Laboratory Animals published by the US National Institutes of Health (8th edition, 2011).

Detailed methods are available in the Supplemental Methods.

### Animal procedures

**Fig. 1:**
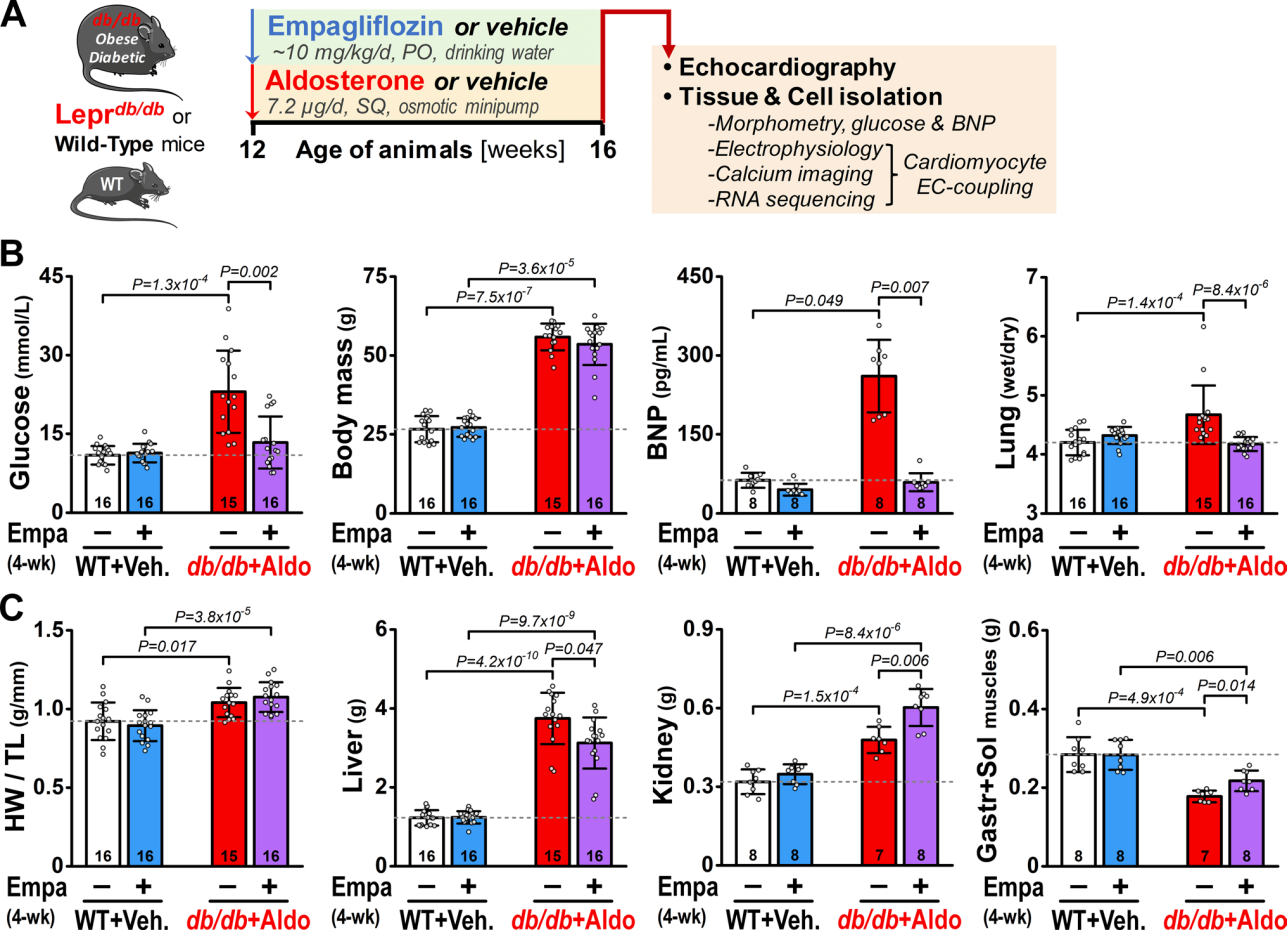
Empagliflozin regulates the multiorgan HFpEF phenotype in obese-diabetic mice. **A** Study protocol of empagliflozin treatment and assessment of multiorgan impairments, cardiac function, and cardiomyocyte excitation–contraction (EC) coupling. Empagliflozin (Empa) was administered in vivo for 4-wks in leptin receptor (Lepr)-deficient *db/db* mice with chronic aldosterone infusion (*db/db* + Aldo) that induced heart failure with preserved ejection fraction (HFpEF) and in vehicle-treated wild-type mice (WT + Veh). **B** Blood glucose, body mass, plasma B-type natriuretic peptide (BNP), and pulmonary edema (quantified as wet-to-dry lung weight) in Empa-treated HFpEF mice. Kruskal–Wallis test with Dunn’s multiple comparison’s test. **C** Cardiac hypertrophy (quantified as heart weight to tibia length ratio, HW/TL), hepatomegaly, kidney weight, and sarcopenia (quantified as combined gastrocnemius and soleus muscle weights) in HFpEF. Welch ANOVA followed by Dunnett’s T3 multiple comparison’s test. The number of animals is shown in the figure

Adult 12-week-old Lepr^db/db^ (Jackson Laboratory, strain #000697) and wild-type (WT) mice, both on the C57BL/6J background, were implanted subcutaneously with osmotic minipumps (Alzet, 2004) that delivered a continuous infusion of either *d*-aldosterone (0.3 μg/h) or vehicle (saline with 5% ethanol) for 4 weeks [17, 23, 36]. The study protocol is shown in Fig. 1A. Mice were kept at standard temperature, humidity, and lighting. Food (Teklad, 2918) and drinking water were provided ad libitum. In line with the common administration route in humans, empagliflozin was administered orally in mice via being dissolved in sterile drinking water (0.033 mg/L, corresponding to 10 mg/kg/d). This dosage has been used in previous preclinical animal studies, and it matches clinically effective plasma concentrations in humans [59]. To improve stability and solubility of empagliflozin, sulfobutylether-β-cyclodextrin (5%) was used in sterile water. Vehicle-treated mice were used as controls. Proper allocation concealment, blinding, and randomization (block randomization with a block size of 4 animals for each genotype and treatment) were used. Each treatment group included equal numbers of male and female animals. No animal was excluded from analysis. One male *db/db* + Aldo mouse (not treated with empagliflozin) died before the conclusion of the study protocol and showed severe pulmonary and systemic congestion by the end of the third week of aldosterone treatment. Data in 8 *db/db* + Aldo and 8 WT + Vehicle mice (both without empagliflozin treatment) have been reported previously when we studied sex differences in the *db/db* + Aldo HFpEF model [36], and were included here as controls. Empagliflozin was similarly effective in both sexes; therefore, the male/female results presented in the article have been merged, but the group comparisons between males and females are provided in Tables S1 and S2.

Animals were injected with heparin (400 U/kg) before the terminal surgical procedure and were subjected to general anesthesia by 2–5% isoflurane inhalation in 100% oxygen throughout. All animals were euthanized by surgical excision of the heart while in deep anesthesia. Enzymatic isolation of left ventricular cardiomyocytes was performed as previously described [21].

### Blood glucose and BNP measurements

Blood glucose levels were measured in blood samples collected from the middle tail vein using the OneTouch UltraMini blood glucose monitoring system and test strips (LifeScan). B-type natriuretic peptide (BNP) levels were measured in blood plasma by ELISA (RayBiotech, EIAM-BNP-1).

### Echocardiography

Transthoracic echocardiography was performed in anesthetized (isoflurane, 0.5–3%) animals. M-mode and Doppler images were acquired using a Vevo 2100 echocardiography system (FUJIFILM VisualSonics) equipped with a 40 MHz transducer.

### Myocyte Ca^2+^ imaging and electrophysiology

Intracellular Ca^2+^ signals were measured in freshly isolated ventricular cardiomyocytes loaded with Fluo-4 AM using confocal microscopy. Action potentials (APs) and ionic currents were recorded in ventricular cardiomyocytes via the patch-clamp technique [36].

### RNA-sequencing

RNA-sequencing from whole-heart lysates was performed by BGI Genomics using paired-end 100 nucleotide sequencing with 40 million reads. Gene expression analysis was performed as previously described [3]. Sequencing data have been deposited in GEO (accession number: GSE285966).

### Protein analysis

Immunoblotting was performed to determine changes in sarcoplasmic reticulum Ca^2+^ ATPase (SERCA), phospholamban (PLN) and CaMKII expression and phosphorylation.

### Statistics

Data are presented as Mean ± SD. Statistical significance of differences was determined using ANOVA followed by appropriate post hoc tests in pairwise comparisons. GraphPad Prism 11 was used for data analysis. *P* < 0.05 was considered statistically significant.

## Results

### Empagliflozin improves multiorgan cardiometabolic HFpEF phenotype

First, we assessed the effect of 4-week in vivo empagliflozin treatment on basic morphometric and metabolic parameters in *db/db* + Aldo HFpEF and vehicle-treated wild-type (WT + Veh) control mice (Fig. 1A). Empagliflozin, a potent antidiabetic agent, normalized high blood glucose levels in *db/db* + Aldo mice (13 ± 5 mmol/L *versus* 23 ± 8 mmol/L), but it did not reduce obesity in *db/db* + Aldo mice (Fig. 1B). Importantly, empagliflozin fully reversed the increased BNP levels and pulmonary congestion (quantified as wet-to-dry lung weight) in *db/db* + Aldo mice, indicating restored cardiac function (Fig. 1B). However, cardiac hypertrophy, quantified as heart weight to tibia length ratio, persisted following empagliflozin treatment in *db/db* + Aldo mice (Fig. 1C). Empagliflozin reduced hepatomegaly in *db/db* + Aldo mice, but it significantly increased kidney weights (Fig. 1C). In HFpEF, there was marked sarcopenia quantified as reduced gastrocnemius and soleus muscle mass, and empagliflozin improved skeletal muscle mass (Fig. 1C). Empagliflozin had no effect on any of these parameters in healthy control mice. These data demonstrate advantageous functional, morphometric, and metabolic extra-cardiac effects of empagliflozin in diabetic HFpEF mice.

### Empagliflozin improves diastolic heart function in HFpEF

**Fig. 2:**
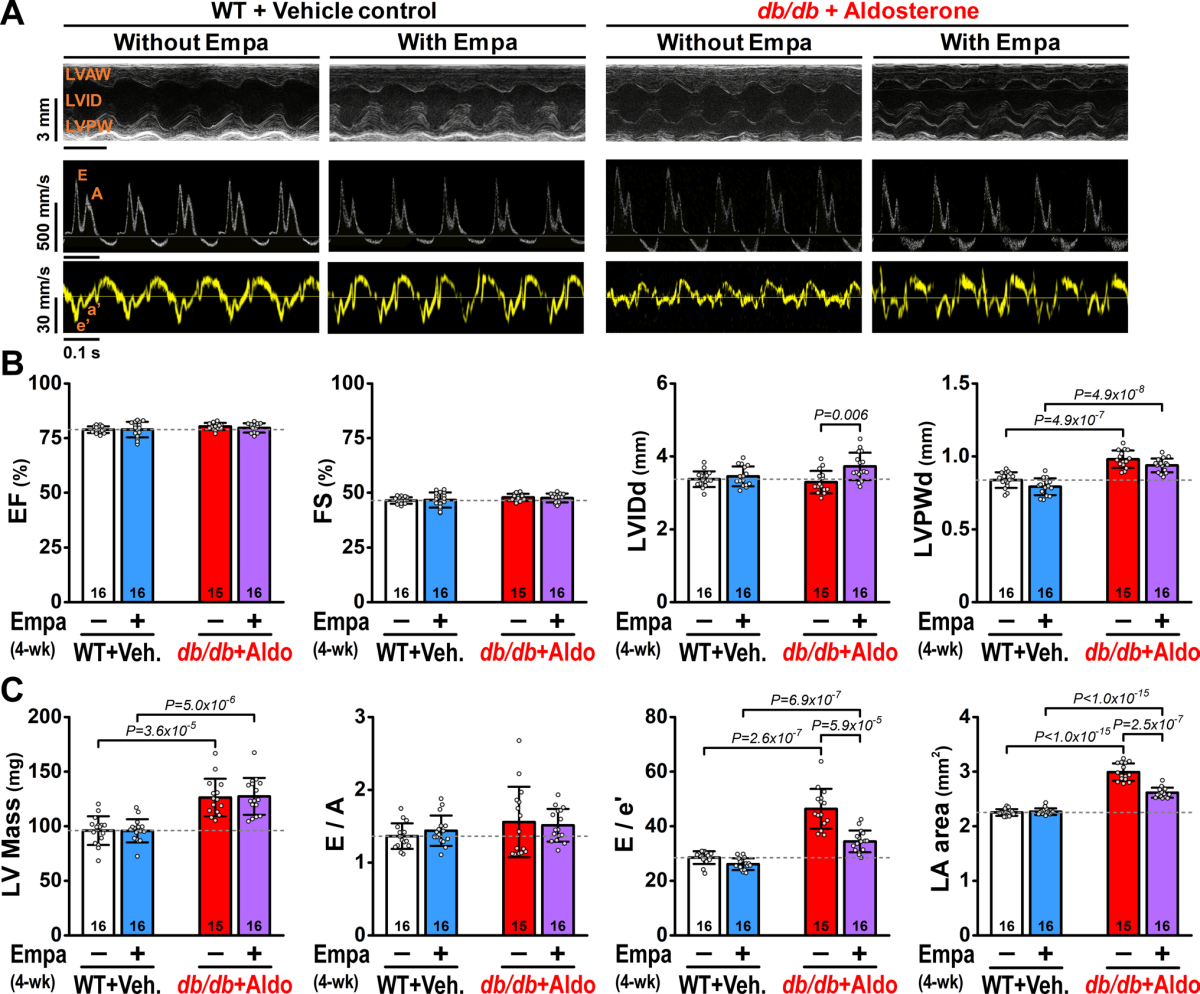
Empagliflozin ameliorates diastolic dysfunction in HFpEF. **A** Left ventricular (LV) M-mode, flow and tissue Doppler echocardiographic images in vehicle-treated wild-type mice (WT + Veh) and in *db/db* mice with chronic aldosterone infusion (*db/db* + Aldo) with or without empagliflozin (Empa) treatment (LVAW, LV anterior wall; LVID, LV internal diameter; LVPW, LV posterior wall). **B** Preserved ejection fraction (EF), fractional shortening (FS), and LV internal diameter at diastole (LVIDd), and thickening of the LV posterior wall at diastole (LVPWd) in Empa-treated HFpEF mice. ANOVA followed by Šídák's multiple comparison’s test. **C** LV mass, diastolic function parameters (E/A, ratio between mitral E wave and A wave; E/e’, ratio between mitral E wave and e’ wave), and enlarged left atria (LA) in Empa-treated HFpEF. Welch ANOVA followed by Dunnett’s T3 multiple comparison’s test. The number of animals is shown in the bar graphs

Echocardiographic evaluation showed preserved ejection fraction (EF) in all treatment groups (Fig. 2A, B) and in both sexes (Table S1). Left ventricular (LV) internal diameter at diastole (LVIDd) was slightly larger in empagliflozin-treated *db/db* + Aldo mice, which may indicate improved LV relaxation and diastolic filling (Fig. 2B). LV posterior wall thickness at diastole (LVPWd) and LV mass were larger in *db/db* + Aldo HFpEF mice, and these echocardiography measures that indicate LV hypertrophy were unchanged in empagliflozin-treated animals (Fig. 2B, C). Diastolic E/A was variable with reduced (in 7/15 mice), (pseudo)normal (in 5/15 mice) or increased (in 3/15 mice) values in *db/db* + Aldo (Fig. 2C), which may indicate a transition in a subset of mice from impaired ventricular relaxation (increasing atrial pressures and reduced E/A) to further ventricular stiffening (causing a so-called restrictive filling pattern with increased E/A) [54]. Empagliflozin treatment in *db/db* + Aldo prevented both the reduction and the increase in E/A (Fig. 2C). Moreover, diastolic E/e’ and left atrial (LA) area were markedly increased in all *db/db* + Aldo HFpEF mice, and empagliflozin significantly attenuated the increases in both parameters (Fig. 2C). These data indicate that in vivo empagliflozin treatment significantly improved diastolic dysfunction in obese-diabetic HFpEF mice.

### Empagliflozin reverses arrhythmogenic action potential changes in HFpEF cardiomyocytes

**Fig. 3:**
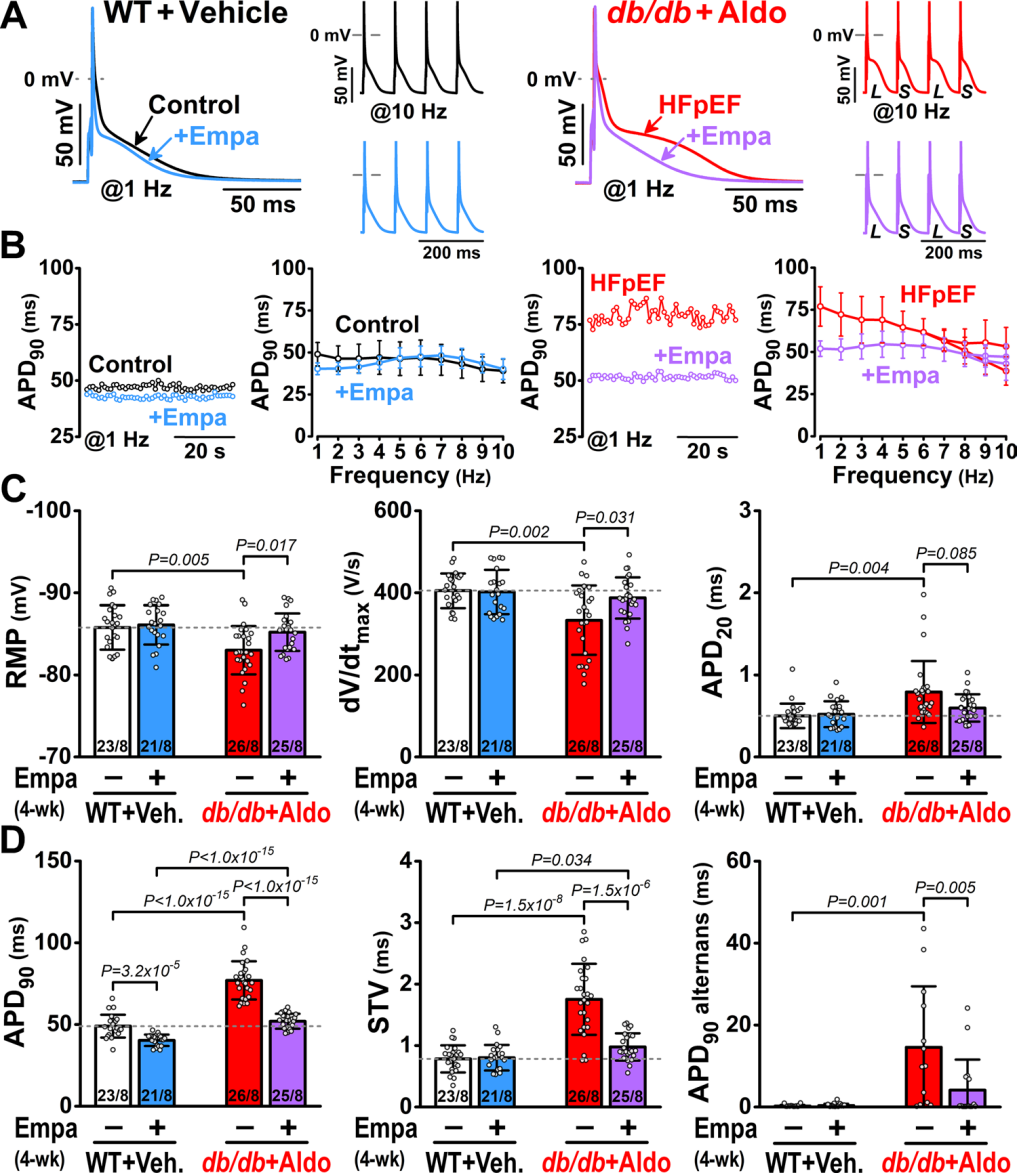
Empagliflozin reverses proarrhythmic action potential changes in HFpEF cardiomyocytes. **A** Ventricular action potentials (APs) in vehicle-treated wild-type mice (WT + Veh) and in *db/db* mice with chronic aldosterone infusion (*db/db* + Aldo) with or without empagliflozin (Empa) treatment. AP duration (APD) alternans (S, short; L, long) at 10 Hz pacing in *db/db* + Aldo. **B** Short-term variability (STV) and frequency-dependence of APD at 90% repolarization (APD_90_). **C** Empagliflozin reversed changes in resting membrane potential (RMP), maximal upstroke velocity (dV/dt_max_), and early AP repolarization (APD_20_) in *db/db* + Aldo. **D** Empagliflozin significantly attenuated the prolongation of APD_90_, decreased STV, and reduced APD_90_ alternans magnitude in *db/db* + Aldo. *n*(cells)/*N*(animals) at 1 Hz pacing, as shown in the bar graphs. For APD_90_ alternans at 10 Hz pacing, *n*(cells)/*N*(animals) = 13/8 for WT + Veh without Empa, 17/8 for WT + Veh with Empa, 13/7 for *db/db* + Aldo without Empa, 16/8 for *db/db* + Aldo with Empa. Welch ANOVA followed by Dunnett’s T3 multiple comparison’s test was used for analyzing all AP parameters except for APD_90_ alternans, where Kruskal–Wallis test with Dunn’s multiple comparison’s test was used

**Fig. 4:**
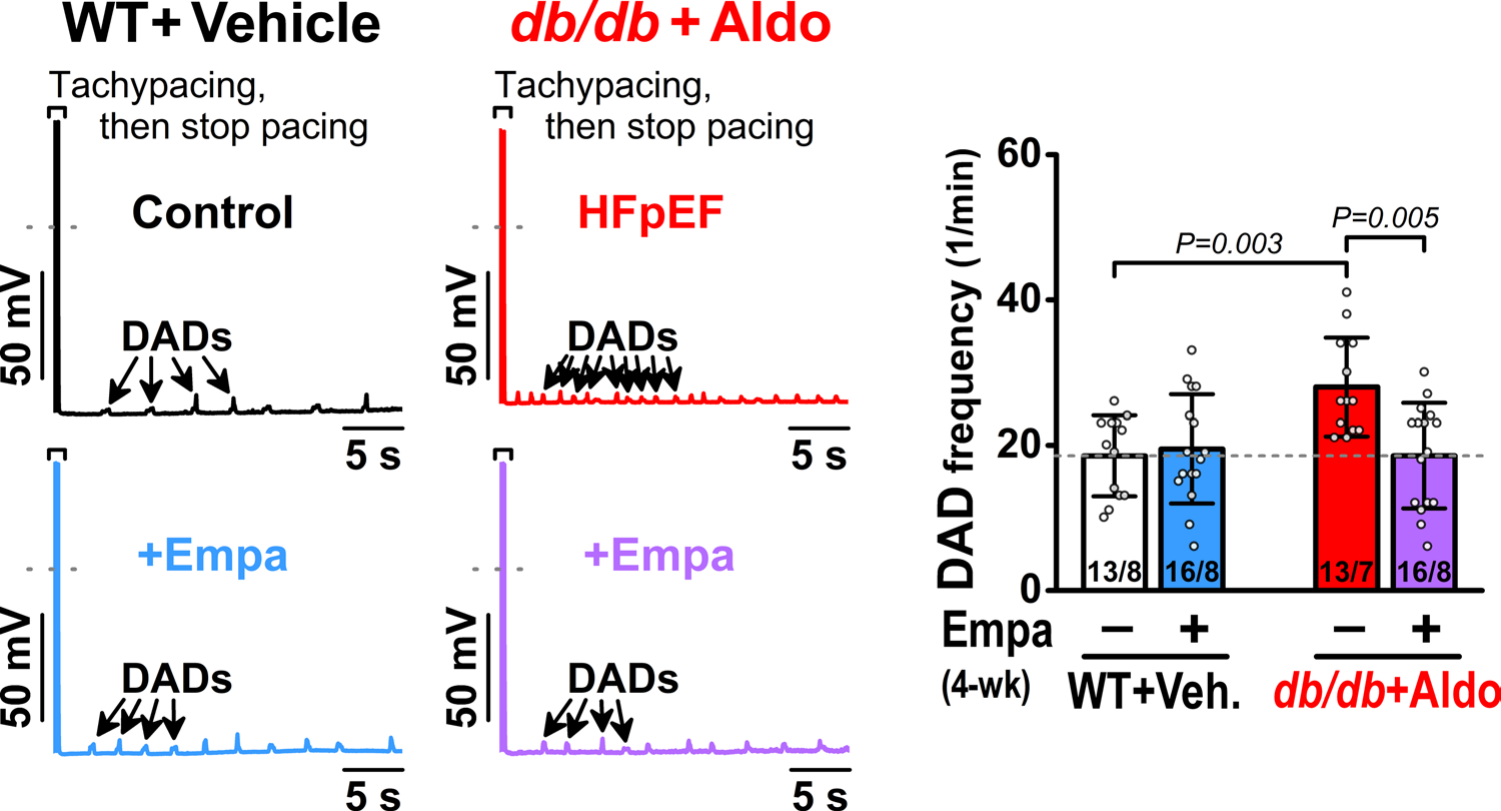
Empagliflozin attenuates delayed afterdepolarizations in HFpEF cardiomyocytes. Delayed afterdepolarizations (DADs) were recorded in ventricular cardiomyocytes following cessation of tachypacing (10 Hz) in vehicle-treated wild-type mice (WT + Veh) and in *db/db* mice with chronic aldosterone infusion (*db/db* + Aldo) with or without empagliflozin (Empa) in vivo treatment. DAD frequency was significantly increased in *db/db* + Aldo, and empagliflozin prevented this increase in DAD frequency in *db/db* + Aldo. *n*(cells)/*N*(animals) is shown in the bar graph. Welch ANOVA followed by Dunnett’s T3 multiple comparison’s test

We then studied ECC mechanisms in HFpEF ventricular cardiomyocytes. First, we measured action potentials (APs) using physiological solutions, temperature, and pacing (Fig. 3). Our prior work had shown that acute pretreatment with empagliflozin could reverse arrhythmogenic AP changes in isolated myocytes from *db/db* + Aldo hearts. For all isolated myocyte experiments here, empagliflozin was completely absent in the extracellular (bath) solution (and during cell isolation), allowing a direct test for a true phenotypic rescue. AP duration at 90% repolarization (APD_90_) was significantly prolonged in *db/db* + Aldo with larger increases at low pacing rates (Fig. 3A, D). Importantly, APD_90_ prolongation was absent in cardiomyocytes from empagliflozin-treated *db/db* + Aldo mice (Fig. 3D). In control myocytes, empagliflozin also slightly shortened APD_90_ (Fig. 3), but it occurred only at low pacing rates (at 1–3 Hz), and no change was found at physiological frequencies (at 6 Hz and above). In *db/db* + Aldo, the depolarized resting membrane potential (RMP), the reduced maximal upstroke velocity (dV/dt_max_), and the prolonged early APD repolarization (APD_20_) were also all reversed in empagliflozin-treated mice, and empagliflozin had no effect on these parameters in healthy controls (Fig. 3C). Short-term variability (STV) of repolarization duration, a sensitive marker for high arrhythmia susceptibility [28, 50], was increased in *db/db* + Aldo, and STV was normalized following empagliflozin treatment (Fig. 3B, D). At high pacing rates (at 8–10 Hz) significant APD_90_ alternans occurred in *db/db* + Aldo, and the alternans magnitude was reduced and threshold frequency was increased following empagliflozin treatment; however, small APD_90_ alternans was still observed at 10 Hz pacing (Fig. 3). The alternans was predominant in AP plateau potentials (without alternation in AP peak or dV/dt_max_) suggesting a Ca^2+^-dependent mechanism rather than reduced Na^+^ channel availability (Fig. 3A); although remodeling in other ionic currents could also contribute to APD_90_ alternans. Moreover, following cessation of tachypacing (10 Hz), *db/db* + Aldo myocytes showed an increase in delayed afterdepolarization (DAD) frequency, which was prevented by in vivo empagliflozin treatment in *db/db* + Aldo mice (Fig. 4).

### Empagliflozin reverses remodeling in major ionic currents in HFpEF cardiomyocytes

**Fig. 5:**
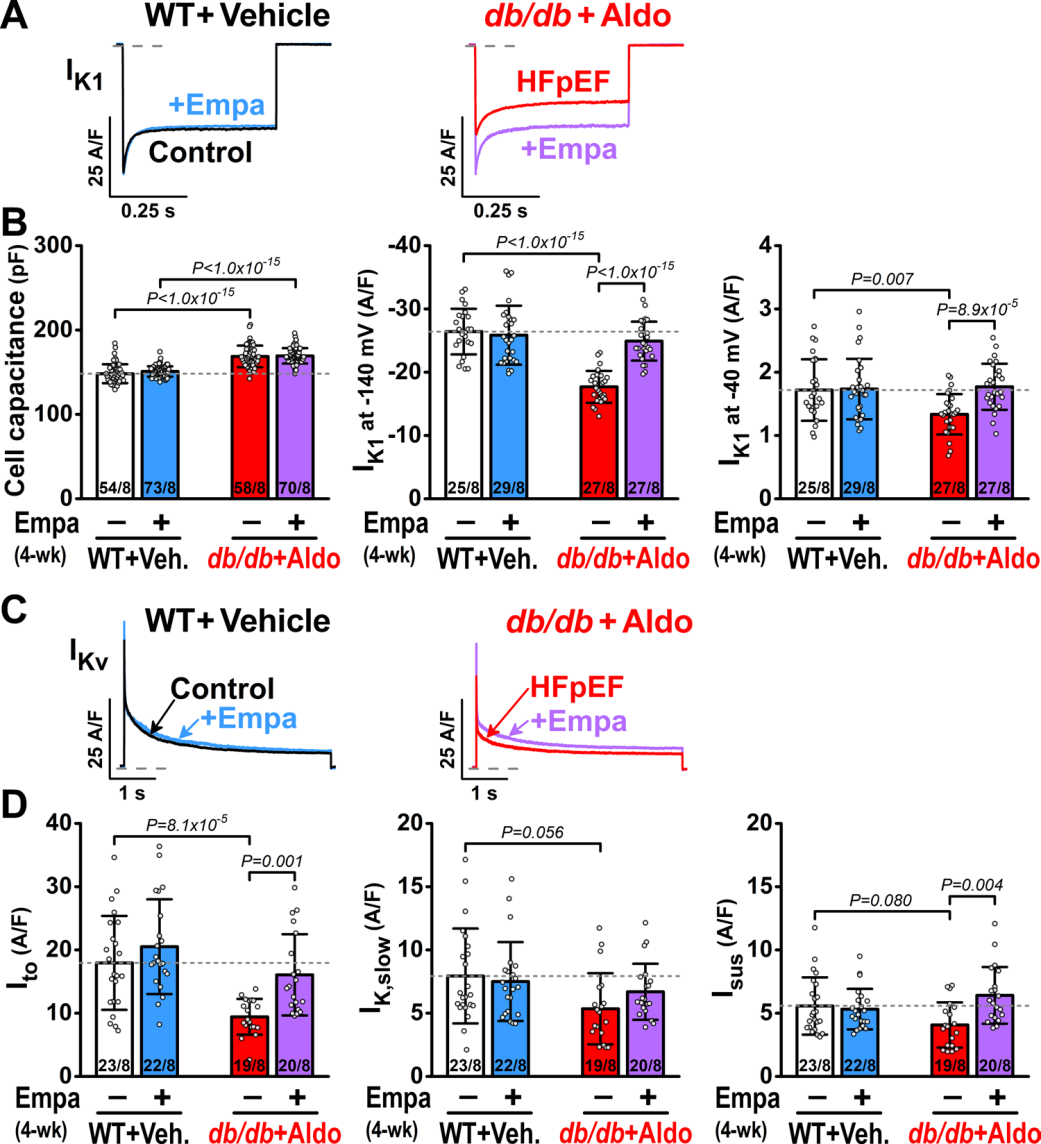
Empagliflozin increases K^+^ currents in HFpEF cardiomyocytes. **A** Inward rectifier K^+^ current (I_K1_) traces at -140 mV in vehicle-treated wild-type mice (WT + Veh) and in *db/db* mice with chronic aldosterone infusion (*db/db* + Aldo) with or without empagliflozin (Empa) treatment. **B** Empagliflozin did not affect cell capacitance, but it increased I_K1_ densities at -140 mV (inward I_K1_) and -40 mV (outward I_K1_) in *db/db* + Aldo. **C** Voltage-gated K^+^ current (I_Kv_) traces. **D** Transient outward K^+^ current (I_to_), slowly inactivating K^+^ current (I_K,slow_), and sustained K^+^ current (I_sus_) were separated by biexponential fitting to I_Kv_ traces. The number of cells/animals is shown in the bar graphs. Welch ANOVA followed by Dunnett’s T3 multiple comparison’s test

We conducted whole-cell voltage-clamp experiments to measure the main voltage-gated ionic currents in cardiomyocytes. To reduce cell-to-cell differences and changes in cell size, we report the magnitude of each ionic current normalized to membrane capacitance, i.e., current density. Consistent with cardiac hypertrophy (Figs. 1C and 2C), cell capacitance was larger in *db/db* + Aldo mice with or without empagliflozin treatment (Fig. 5B).

We measured the major K^+^ currents of the murine ventricular myocytes. The density of the inward rectifier K^+^ current (I_K1_) was reduced both in the inward (at -140 mV) and outward (-40 mV) directions in *db/db* + Aldo, and these changes were reversed by empagliflozin (Fig. 5A, B). The reduced I_K1_ in HFpEF can depolarize the resting membrane potential and increase the propensity for triggered beats. The voltage-gated K^+^ currents (I_Kv_) were separated using biexponential fitting to the current decay. The transient outward K^+^ current (I_to_) density was markedly reduced in *db/db* + Aldo in line with prolonged APD_20_ (Fig. 3C), and I_to_ was increased by empagliflozin treatment (Fig. 5C, D). The slowly inactivating K^+^ current (I_K,slow_) and the non-inactivating sustained K^+^ current (I_sus_) densities were reduced (less than I_to_) in *db/db* + Aldo, and the reductions in the currents were prevented by empagliflozin treatment in *db/db* + Aldo (Fig. 5C, D).

**Fig. 6:**
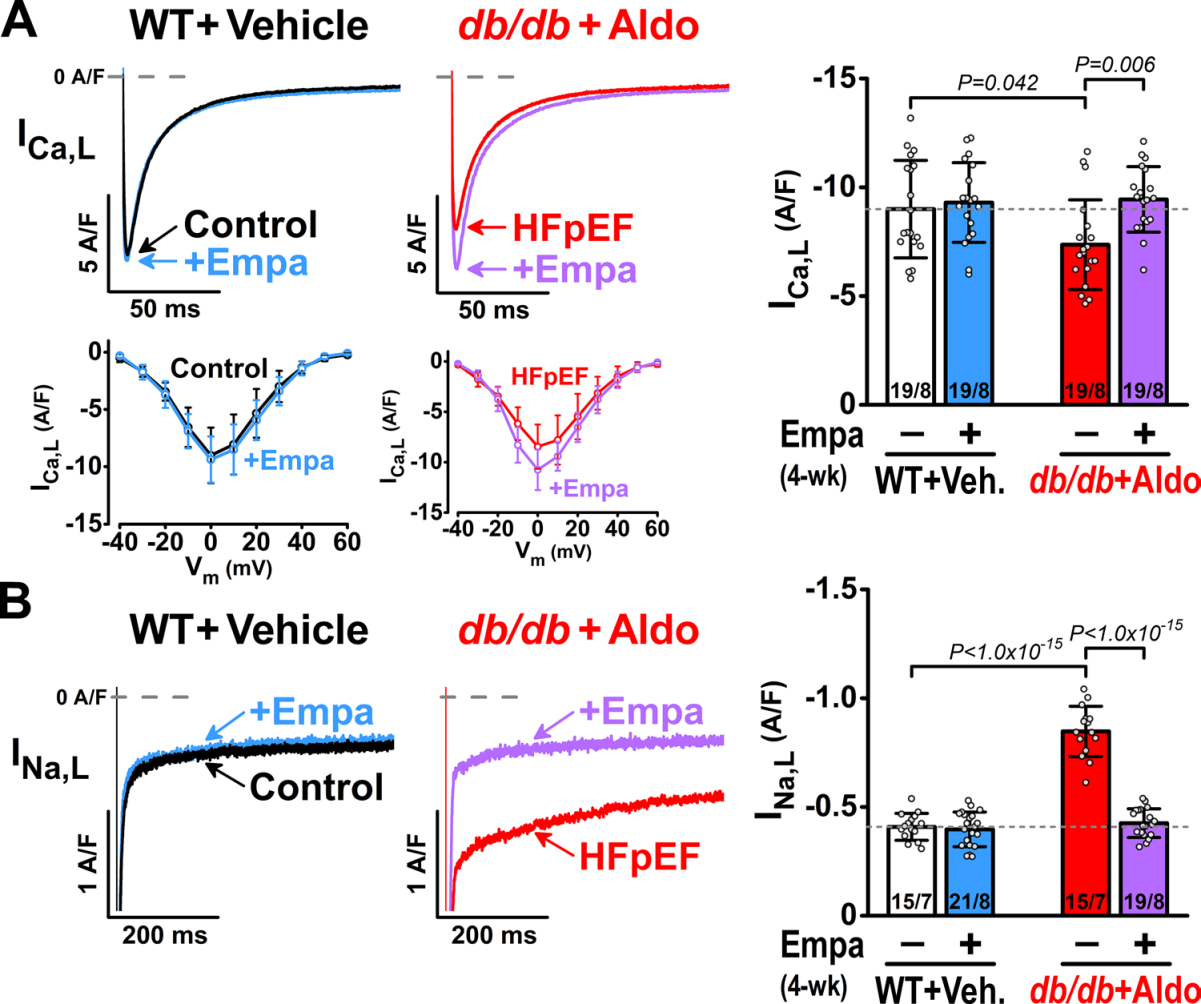
Empagliflozin reduces the late Na^+^ current and increases the L-type Ca^2+^ current in HFpEF cardiomyocytes. **A** L-type Ca^2+^ current (I_Ca,L_) traces at 0 mV in vehicle-treated wild-type mice (WT + Veh) and in *db/db* mice with chronic aldosterone infusion (*db/db* + Aldo) with or without empagliflozin (Empa) treatment. I_Ca,L_ was slightly reduced in *db/db* + Aldo and empagliflozin increased I_Ca,L_ in *db/db* + Aldo. **B** Late Na^+^ current (I_Na,L_) traces at -40 mV. Empagliflozin reduced the enhanced I_Na,L_ in *db/db* + Aldo to control. Number of cells/animals is shown in the bar graphs. ANOVA followed by Šídák's multiple comparison’s test

Next, we measured the cardiomyocytes’ major depolarizing voltage-gated currents, the L-type Ca^2+^ current (I_Ca,L_) and the late Na^+^ current (I_Na,L_). I_Ca,L_ density was reduced in *db/db* + Aldo, and empagliflozin increased I_Ca,L_ back to control level (Fig. 6A). Empagliflozin had no effect on I_Ca,L_ voltage-dependence and no effect on I_Ca,L_ in control (Fig. 6A). I_Na,L_, a tiny current in healthy cardiomyocytes, was markedly increased in *db/db* + Aldo, and empagliflozin treatment fully reversed I_Na,L_ enhancement while not affecting I_Na,L_ in control (Fig. 6B). These data indicate that in vivo empagliflozin treatment provides significant functional electrophysiological benefits by reversing all major ionic current changes in HFpEF cardiomyocytes.

### Empagliflozin reverses prolonged Ca^2+^ transients decay in male HFpEF cardiomyocytes

**Fig. 7:**
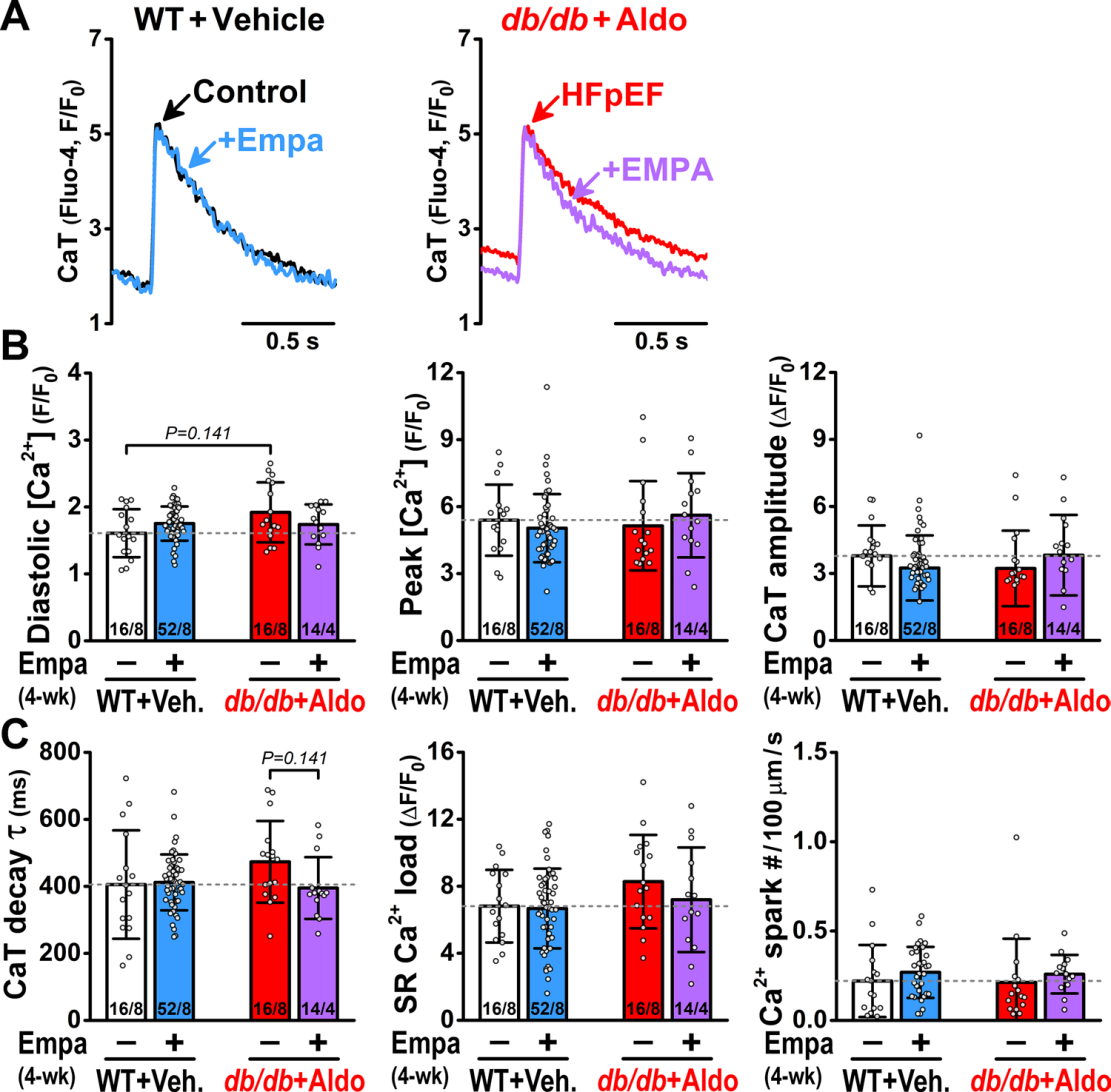
Intracellular Ca^2+^ handling following empagliflozin treatment in HFpEF cardiomyocytes. **A** Intracellular Ca^2+^ signals in vehicle-treated wild-type mice (WT + Veh) and in *db/db* mice with chronic aldosterone infusion (*db/db* + Aldo) with or without empagliflozin (Empa) treatment. Cardiomyocytes were paced at 1 Hz. **B** Intracellular Ca^2+^ transient (CaT) parameters. Diastolic [Ca^2+^] is the ratio of minimum F between beats at steady-state 1 Hz pacing and the resting F_0_. Welch ANOVA followed by Dunnett’s T3 multiple comparison’s test. **C** CaT decay tau, sarcoplasmic reticulum (SR) Ca^2+^ content (assessed by rapid local application of 10 mmol/L caffeine), and Ca^2+^ spark rate. Number of cells/animals is shown in the bar graphs. For Ca^2+^ sparks data, *n*(cells)/*N*(animals) = 16/8 for WT + Veh without Empa, 32/8 for WT + Veh with Empa, 16/8 for *db/db* + Aldo without Empa, and 14/4 for *db/db* + Aldo with Empa. Kruskal–Wallis test followed by Dunn’s multiple comparison’s test

We then measured intracellular Ca^2+^ transients (CaT) in paced myocytes. All CaT parameters were relatively preserved in *db/db* + Aldo (Fig. 7), with trends towards slightly increased diastolic [Ca^2+^] and longer CaT decay in *db/db* + Aldo (Fig. 7). We previously showed that when cells from male and female mice were analyzed separately, both CaT decay tau and diastolic [Ca^2+^]_i_ during pacing were significantly increased only in male *db/db* + Aldo HFpEF mice [36]. We found similar male-specific CaT effects here in *db/db* + Aldo myocytes that were largely normalized in empagliflozin-treated male *db/db* + Aldo mice (CaT decay taus for WT + Vehicle, *db/db* + Aldo, and *db/db* + Aldo + Empagliflozin were 385 ± 127, 582 ± 98, and 388 ± 98 ms, respectively; Table S2) and that contrasts with females, where the same group of CaT decay taus were little altered by HFpEF or HFpEF + Empa (426 ± 198,408 ± 82 and 422 ± 78 ms, respectively). Therefore, this is one point where the sex difference was apparent [36]. Diastolic Ca^2+^ spark rate was unchanged in *db/db* + Aldo, and empagliflozin did not affect Ca^2+^ spark rate in control or in HFpEF (Fig. 7C).

### Empagliflozin shifts cardiac transcriptomic profile in HFpEF

**Fig. 8:**
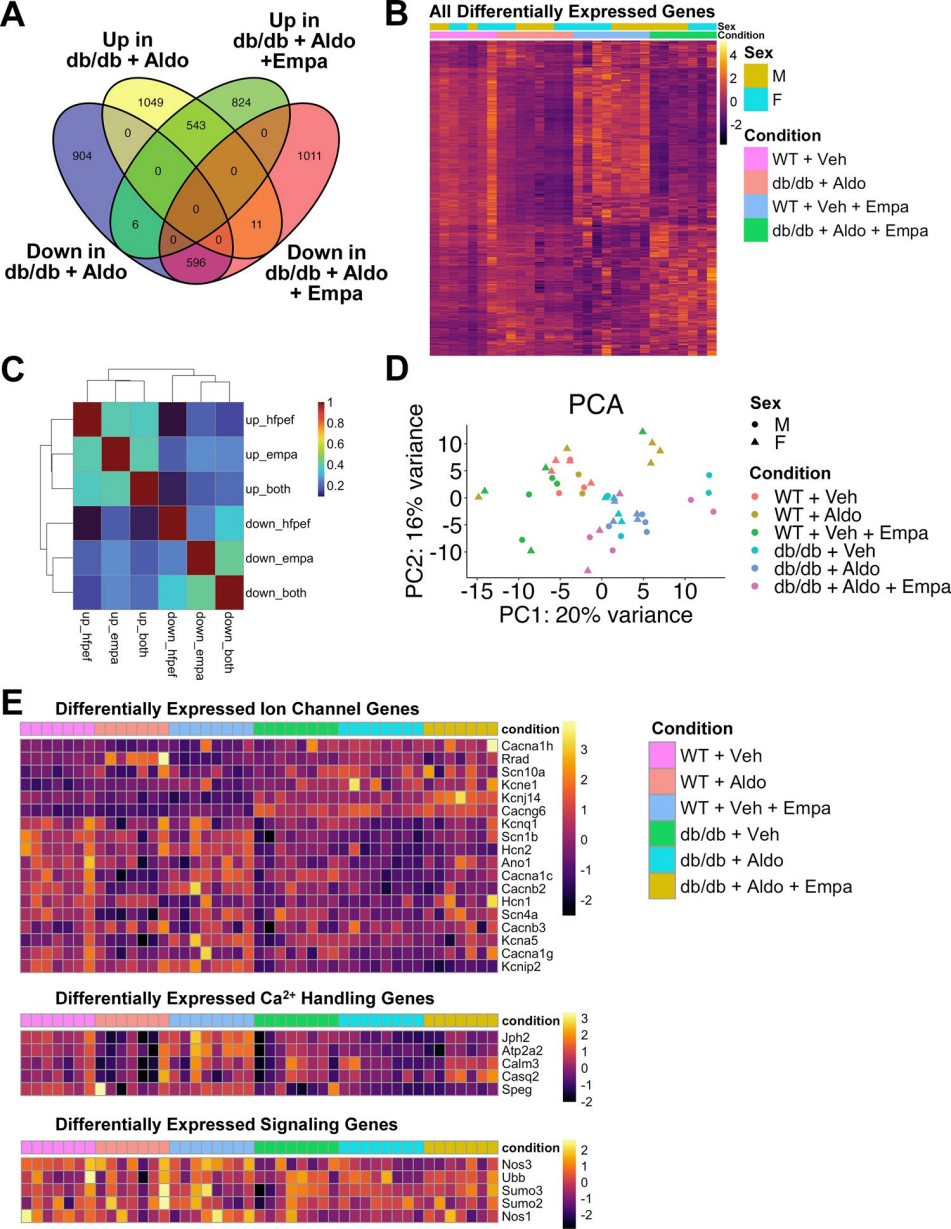
Empagliflozin alters cardiac gene expression profile in HFpEF. **A** Venn diagram of genes for the upregulated and downregulated differentially expressed genes in *db/db* mice with chronic aldosterone infusion (*db/db* + Aldo) HFpEF hearts with and without empagliflozin (Empa) treatment. **B** Normalized gene expression of all genes differentially expressed in vehicle-treated wild-type (WT + Veh) control mice and in *db/db* + Aldo with or without Empa treatment color-coded by condition and sex (M, male; F, female). Genes are clustered by hierarchical clustering using Euclidean distance. **C** Heatmap showing pairwise Jaccard similarity index of shared Gene Ontology/Biological Processes (GO:BP) term enrichment between differentially expressed genes (*P* < 0.05) in each comparison. **D** Principal component analysis (PCA) of whole-heart transcriptomes reveals distinct clusters in different treatment groups. **E** Differentially expressed genes for select ion channels, Ca^2+^ handling and signaling proteins. WT + Aldo, wild-type mice with chronic aldosterone infusion. *N*(animals) = 7 for WT + Veh, 8 for WT + Veh + Empa, 7 for WT + Aldo, 8 for *db/db* + Veh, 8 for *db/db* + Aldo, and 7 for *db/db* + Aldo + Empa. Significance threshold was determined by the 5% false discovery rate adjusted for multiple comparisons by the Benjamini–Hochberg method. Z-scores are shown in the color map

Empagliflozin reversed the changes in ionic currents and CaT even without empagliflozin being present in the extracellular perfusion solution during the recordings. Since ion channels and Ca^2+^ handling proteins are regulated at the transcription level in the failing heart, we performed RNA-sequencing in whole-heart lysates in *db/db* + Aldo and control mice with and without in vivo empagliflozin treatment. Cardiac gene expression in *db/db* + Aldo is markedly different from WT + Vehicle control with 1603 upregulated and 1506 downregulated genes (Fig. 8A, Online Resource 1). In the second batch of sequencing, empagliflozin-treated *db/db* + Aldo mice had similar degrees of gene expression changes versus empagliflozin-treated controls, with 1373 upregulated and 1618 downregulated genes (Fig. 8A, Online Resource 2). 543 genes were upregulated *versus* control and 596 were downregulated *versus* control in both experiments (Fig. 8A). There were sex-specific differences in gene expression. Among differentially expressed genes in *db/db* + Aldo, only 24.4% were shared between males and females, 13.9% were specific to males and 61.8% were specific to females (Fig. S1A, Online Resource 3). However, following empagliflozin treatment in *db/db* + Aldo, 74.6% of differentially expressed genes were specific to males, 14.78% were shared in both sexes, and 10.7% were specific to females (Fig. S1B, Online Resource 3). Analysis of batch-normalized gene expression between experiments showed that empagliflozin treatment did not reverse the gene expression changes in *db/db* + Aldo to control in either sex (Fig. 8B). In addition, gene ontology analysis of differentially expressed genes that were either upregulated or downregulated in one or both experiments revealed similar overall changes in functional pathways regardless of empagliflozin treatment or sex (Fig. 8C, S1C, and Online Resource 4–5). Nonetheless, some pathways were responsive to empagliflozin treatment. Empagliflozin rescued the downregulated Notch signaling pathway and upregulated peroxisome proliferator-activated receptor (PPAR) and phosphoinositide-3-kinase (PI3K)–Akt signaling pathways, as well as multiple metabolic pathways in *db/db* + Aldo (Figs. S2 and S3, Online Resource 4). Among pathways directly affecting cardiomyocyte ECC, empagliflozin upregulated the calcium signaling pathway in *db/db* + Aldo; however, it did not restore the downregulated cAMP signaling pathway (Figs. S2 and S3, Online Resource 4).

Pathway analysis also revealed sex differences. In female *db/db* + Aldo hearts, motor proteins and arachidonic acid metabolism were downregulated, and these changes were rescued by empagliflozin (Fig. S4, Online Resource 5). In contrast, in *db/db* + Aldo males, mitogen-activated protein kinase (MAPK) signaling was among the most prominent downregulated pathways and remained suppressed following empagliflozin treatment, unlike PI3k–Akt and NOD-like receptor signaling which were rescued by empagliflozin treatment (Fig. S4, Online Resource 5). Empagliflozin also reversed the upregulation of genes associated with reactive oxygen species (ROS) in *db/db* + Aldo males (Fig. S5, Online Resource 5). Interestingly, in *db/db* + Aldo females, empagliflozin upregulated hypoxia-inducible factor-1 (HIF-1) signaling (Fig. S5, Online Resource 5).

To better characterize the individual impacts of (*a*) *db/db* genetic background, (*b*) aldosterone, and (*c*) empagliflozin treatment on the gene expression patterns in these animals, we compared RNA expression profiles of hearts from non-treated, aldosterone-treated, and empagliflozin-treated control and *db/db* mice. Principal component analysis of batch-corrected RNA expression revealed that samples largely clustered separately based on control versus *db/db* genotype (Fig. 8D). Thus, many downregulated key ion channel and Ca^2+^ handling genes in *db/db* + Aldo (*versus* WT + Vehicle) were also downregulated in empagliflozin-treated *db/db* + Aldo (*versus* empagliflozin-treated WT + Vehicle) hearts (Fig. 8E), including Cacna1c (Ca_V_1.2), Kcnip2 (KChIP2), Atp2a2 (SERCA2), Jph2 (Junctophilin-2), and Speg (Striated muscle preferentially expressed protein kinase). However, empagliflozin treatment in *db/db* + Aldo reversed a subset of the downregulated genes, including Scn4a (Na_V_1.4), Scn1b (Na_V_β_1_), Cacnb2 (subunit of Ca_V_β_2_), Kcnq1 (K_V_7.1), and Ano1 (Anoctamin-1, Ca^2+^-activated Cl^−^ channel). In contrast to the downregulated genes, a majority of the upregulated ion channel and Ca^2+^ handling genes in *db/db* + Aldo reversed following empagliflozin treatment (Fig. 8E), including Scn10a (Na_V_1.8), Kcna5 (K_V_1.5), Kcne1 (MinK), Cas2 (Calsequestrin 2), and Calm3 (Calmodulin 3). However, other genes remained upregulated in *db/db* + Aldo following empagliflozin treatment, including Rrad (Rad), Cacna1h (Ca_V_3.2), and Cacng6 (Ca_V_γ_6_). Interestingly, empagliflozin treatment in *db/db* + Aldo markedly increased the expression of pacemaker channels Hcn1 and Hcn4 (Fig. 8E). These data suggest that the functional improvements in ionic currents and Ca^2+^ handling in empagliflozin-treated *db/db* + Aldo cardiomyocytes were not simply due to transcriptional changes but rather post-translational regulatory mechanisms and protein stability. In line with that, Nos1 (nitric oxide synthase 1, neuronal) was downregulated in *db/db* + Aldo and empagliflozin treatment rescued Nos1 expression (Fig. 8E). Prkg1 (protein kinase G1, PKG1) expression is also increased in empagliflozin-treated *db/db* + Aldo hearts (Online Resource 1–2). Importantly, Slc5a2 (SGLT2) expression was very low or undetectable in whole-heart lysates from all experimental groups, suggesting that extra-cardiac (i.e., better systemic glucose control) and/or off-target effects of empagliflozin could mediate functional benefits in HFpEF hearts.

### Empagliflozin-dependent unique gene clusters in HFpEF

**Fig. 9:**
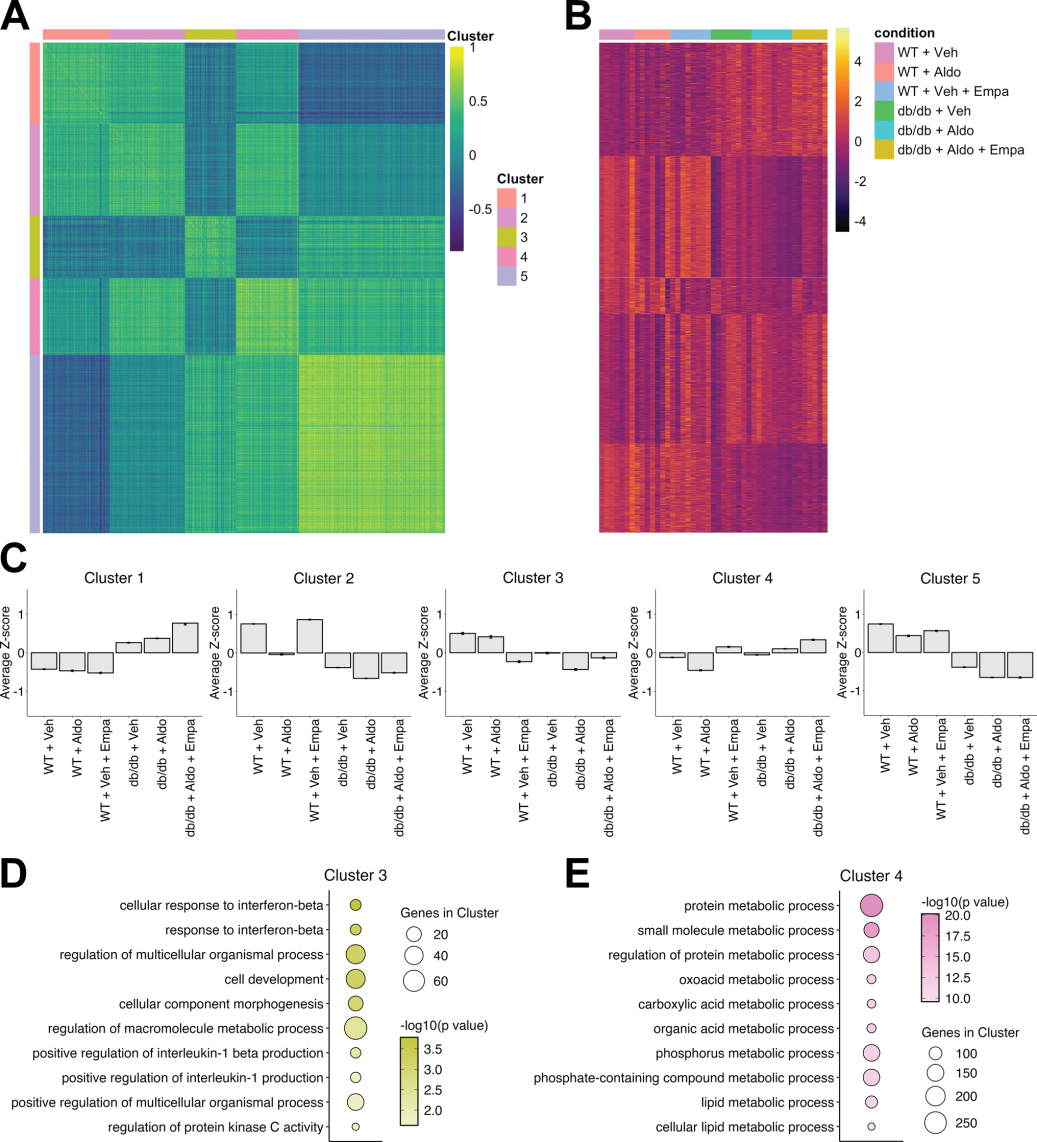
Functional analysis of differentially expressed HFpEF genes. **A** Pairwise gene–gene spearman correlations for all differentially expressed genes (DEGs) color-coded by k-nearest-neighbor (KNN) clustering. **B** Normalized gene expression of all DEGs color-coded by condition and KNN cluster. **C** Average Z-scores of genes within each cluster by condition. **D, E** Enrichment of Gene Ontology (GO) biological processes based on genes in clusters 3 (**D**) or cluster 4 (**E**). *N*(animals) = 7 for WT + Veh, 8 for WT + Veh + Empa, 7 for WT + Aldo, 8 for *db/db* + Veh, 8 for *db/db* + Aldo, and 7 for *db/db* + Aldo + Empa. Color of dot represents Fisher exact *P* value after Benjamini–Hochberg adjustment for multiple comparisons. Size of dot is proportional to the number of differentially expressed genes in that category. WT, wild-type; Veh, vehicle; Aldo, aldosterone; Empa, empagliflozin; *db/db*, leptin receptor-deficient mouse

Functional gene modules were identified by K-nearest neighbor (KNN) clustering of gene–gene expression correlation values for genes which were differentially expressed between control and HFpEF samples (Fig. 9A, Online Resource 6). This revealed 5 clusters of genes with varied expression patterns amongst the different biological conditions (Fig. 9A, B). Notably, clusters 1 and 5 captured expression changes driven by the *db/db* genotype, cluster 2 captured gene expression changes with aldosterone-dependence, and clusters 3 and 4 captured gene expression changes with empagliflozin-dependence (Fig. 9C). Cluster 3 contained genes generally downregulated with empagliflozin treatment which were enriched for functions including processes promoting inflammation and protein kinase C (PKC) activation (Fig. 9D, Online Resource 7). Cluster 4 contained genes generally upregulated with empagliflozin treatment which were enriched for functions including lipid and protein metabolism (Fig. 9E, Online Resource 7). Importantly, clusters 3 and 4 also included multiple genes in ubiquitin and SUMO pathways (e.g., Ubb, Usp2, Ube2g1, Ube2e3, Ufd1, Sumo2, and Sumo3) and ER-stress (e.g., Kdelr1, Herpud1, and Txndc12) regulating protein processing, stability, and localization. Cluster 4 also included multiple fibrosis-related genes upregulated in HFpEF and reversed by empagliflozin treatment (e.g., Col1a1, Col1a2, Col5a1, Col5a2, and Col6a1). Among differentially expressed ion channel and Ca^2+^ handling genes, clusters 1 and 5 (*db/db*-dependent) included Speg, Kcnip2, Rrad, Scn10a, and Kcne1; cluster 2 (aldosterone-dependent) included Jph2, Atp2a2, Cacna1c, Cacnb2, and Kcnq1; and clusters 3 and 4 (empagliflozin-dependent) included Casq2, Calm3, Kcna5, Scn4a, and Hcn1 (Online Resource 6).

Overall, there was a complex pattern of gene expression change following empagliflozin treatment with accentuation of existing differences rather than a reversal of the gene expression profile in *db/db* + Aldo toward control values. This could have two implications: (1) different transcriptomic profiles can result in normal physiological heart function and (2) the observed electrophysiological and ECC differences are primarily mediated by regulatory post-translational modifications and protein stability/recycling. This is in line with the concept of multiple “good enough solutions”, i.e., combinations of individual changes in biological systems can be balanced in different ways and can produce an equivalent outcome to accomplish normal biological functions [61].

### Empagliflozin effects on key ECC protein expression and phosphorylation in HFpEF

**Fig. 10:**
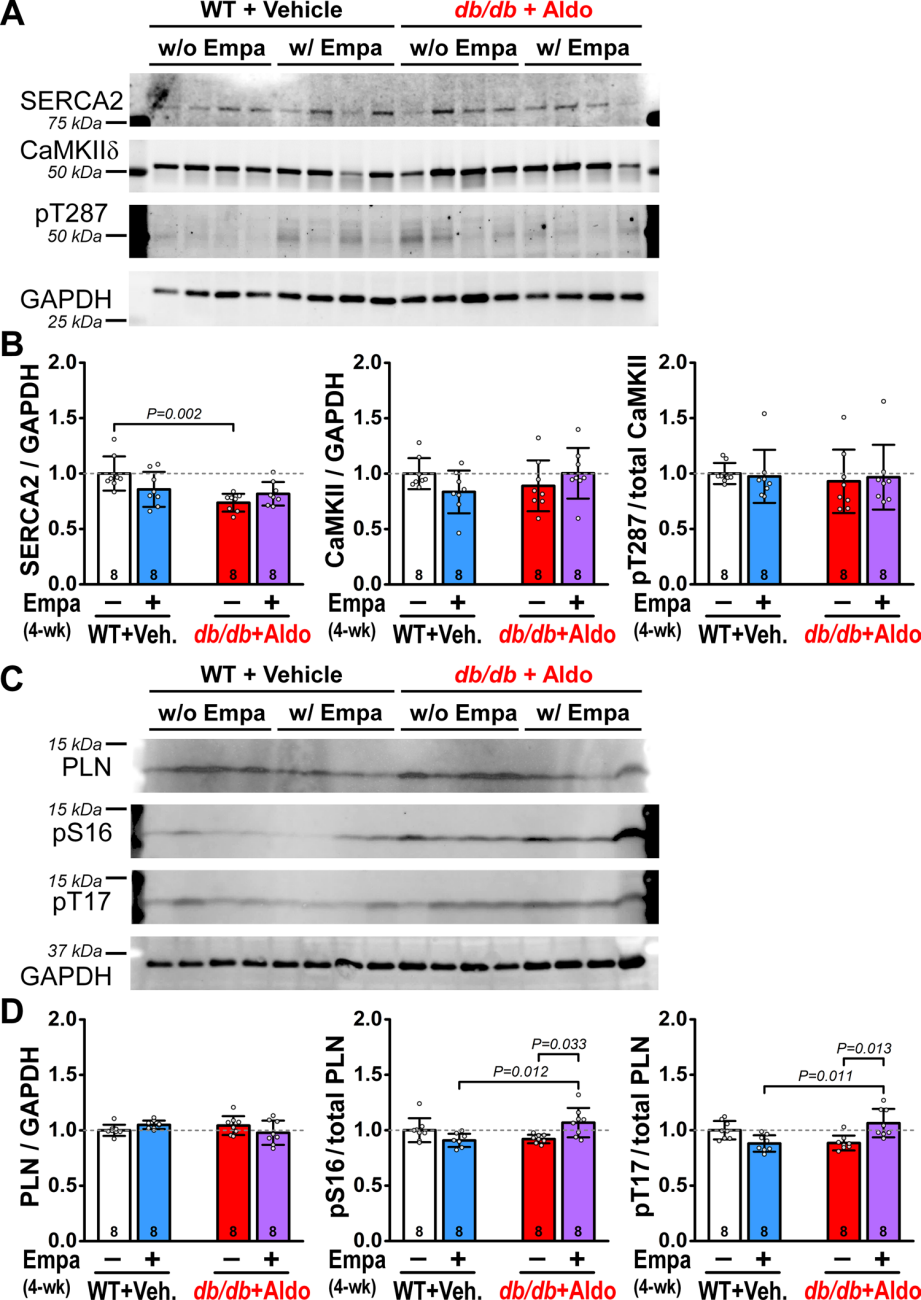
Expression and phosphorylation of key Ca^2+^ handling proteins in HFpEF hearts. **A** Sarcoplasmic reticulum Ca^2+^ ATPase 2 (SERCA2) expression, and Ca^2+^/calmodulin-dependent protein kinase II (CaMKII) expression and phosphorylation at the threonine 287 (T287) site. **B** Reduced SERCA2 expression in *db/db* + Aldo without empagliflozin (Empa) treatment, and unchanged CaMKII expression and phosphorylation. **C** Phospholamban (PLN) expression and phosphorylation at serine 16 (S16) and threonine 17 (T17) sites. **D** Unchanged PLN expression but increased S16 and T17 phosphorylation in Empa-treated *db/db* + Aldo hearts. Protein expression was normalized to WT + Vehicle. The number of animals is shown in the bar graphs. Kruskal–Wallis test followed by Dunn’s multiple comparison’s test. WT, wild-type; Veh, vehicle; Aldo, aldosterone; Empa, empagliflozin; *db/db*, leptin receptor-deficient mouse

Key ECC proteins are importantly regulated by post-translational modifications. Because empagliflozin reversed the male-specific prolonged CaT decay (Fig. 7, Table S2) in *db/db* + Aldo, we measured protein expression and phosphorylation of SERCA2 and phospholamban (PLN). SERCA2 protein expression was reduced in *db/db* + Aldo mirroring RNA results (Fig. 10A, B). PLN expression was unchanged in *db/db* + Aldo with or without empagliflozin treatment (Fig. 10C, D). Phosphorylation of PLN, which relieves its inhibitory action on SERCA2, was increased in the empagliflozin-treated *db/db* + Aldo hearts both at the predominantly PKA-specific serine 16 (pS16) and CaMKII-specific threonine 17 (pT17) sites (Fig. 10C, D). Interestingly, unlike in HFrEF, CaMKII expression and autophosphorylation (pT287) were unchanged in *db/db* + Aldo hearts (Fig. 10A, B). These results suggest SERCA downregulation and altered PLN phosphorylation might be key mediators of altered Ca^2+^ handling in *db/db* + Aldo myocytes, particularly in male *db/db* + Aldo mice (Table S3). CaMKII activation via other post-translational modifications, e.g., oxidation, *S*-nitrosylation, and *O*-GlcNAcylation, may play important roles in obese-diabetic HFpEF, as our prior work has suggested in hyperglycemia and diabetes [21].

## Discussion

### Multiorgan effects of empagliflozin in HF and diabetes

Empagliflozin, an SGLT2 inhibitor, provides unparalleled benefit to HF patients, not seen with other antidiabetic agents [58]. Empagliflozin via SGLT2 inhibition has advantageous metabolic, hemodynamic, and renal effects in diabetes by reducing elevated blood glucose levels [12, 58]. However, empagliflozin also provides similar cardiovascular benefits in HF patients without T2DM [1]. Empagliflozin has cardioprotective effects even in isolated cardiomyocytes (in which SGLT2 expression is minimal or lacking) and in SGLT2 knockout animals, indicating potentially beneficial off-target effects [15]. Multiple mechanisms have been suggested to mediate these cardioprotective effects of empagliflozin directly in cardiomyocytes; however, the exact molecular target(s) remain unclear [35]. Here, we tested empagliflozin for reversing arrhythmogenic ECC and ion channel remodeling in a two-hit murine model of cardiometabolic HFpEF [23]. We found that chronic in vivo empagliflozin treatment not only normalized blood glucose levels (Fig. 1) and attenuated diastolic dysfunction (Fig. 2) but also rescued HFpEF-induced changes in APs (Fig. 3) and DADs (Fig. 4), ionic currents (Figs. 5 and 6), and diastolic Ca^2+^ handling (Fig. 7 and Table S2), the latter being characteristic of male HFpEF hearts [36].

Empagliflozin slightly reduced body weight and cardiac hypertrophy in diabetic patients [1, 57] and in obese-diabetic ZSF1 rats [51]. However, empagliflozin did not reduce body weight, heart weight, LV mass, or cardiomyocyte capacitance in *db/db* + Aldo mice (Figs. 1, 2 and 5). The lack of this effect might be due to the shorter (4-week) treatment duration, unrestricted access to food, and alterations in body composition in *db/db* + Aldo mice, including reduced liver weight, but increased kidney weight and skeletal muscle weight in empagliflozin-treated HFpEF mice (Fig. 1). Empagliflozin improved cardiovascular outcomes in patients with HFpEF regardless of baseline body mass index (BMI), indicating that attenuation of obesity might not be the primary mechanism for its benefit [1]. Empagliflozin was shown to increase kidney weight by inducing cell hypertrophy in the distal segments of proximal tubules and the collecting duct (cells which do not express SGLT2), as a potential compensatory mechanism increasing reabsorption of glucose, NaCl, and H_2_O [56]. The improved cardiac function may enhance exercise tolerance and promote improvements in skeletal muscle contractility and mitochondrial function, as demonstrated in ZSF1 rats [63]. Empagliflozin also improved cardiac mitochondrial function in ZSF1 rats in part by increasing cardiolipin levels [51]. Empagliflozin reduced mitochondrial ROS in diabetic rat atrial myocytes [30] through a mechanism that requires further investigation in HFpEF ventricular myocytes.

### Antiarrhythmic effects of empagliflozin in HF and diabetes

Antiarrhythmic effects of empagliflozin have previously been reported in patients with T2DM and HFrEF [7, 8, 42]; however, further studies are needed in HFpEF. In diabetic patients, empagliflozin reduced QTc and dispersion of repolarization (Tpeak-to-Tend, TpTe), and reduced new-onset ventricular arrhythmias following acute myocardial infarction [7, 8, 42]. In HFrEF patients, dapagliflozin, another SGLT2 inhibitor, reduced the risk of ventricular arrhythmia, cardiac arrest, or sudden death [13], and it also reduced QTc and dispersion of repolarization [40]. In transverse aortic constriction-induced HFrEF mice, empagliflozin rescued APD prolongation and impairments in late Na^+^ current and Ca^2+^ handling, and attenuated arrhythmia susceptibility [46, 62]. Empagliflozin also shortened drug-induced long QT intervals, APD_90_, and decreased VT burden in Langendorff-perfused rabbit hearts [64]. Here, we showed that chronic in vivo empagliflozin treatment in *db/db* + Aldo reversed APD_90_ prolongation and attenuated APD_90_ alternans, and also reversed the increases in STV and DAD (Figs. 3 and 4). In addition to ventricular arrhythmias, HFpEF is frequently associated with atrial fibrillation (AF) [45]. Empagliflozin reduced left atrial enlargement in *db/db* + Aldo mice (Fig. 2), potentially indicating reduced mechanical stress and atrial remodeling in HFpEF, and these mechanisms may contribute to reduced AF susceptibility. In line with this, SGLT2 inhibitors reduced AF risk in patients with T2DM [31]. Interestingly, dapagliflozin may have a more pronounced AF reducing effect than empagliflozin in both patients [34] and preclinical animals [64].

Dapagliflozin was shown to acutely inhibit peak I_Na_ preferentially in human atrial cardiomyocytes and atrial-like human-induced pluripotent stem cell-derived cardiomyocytes (hiPSC-CMs) with an IC_50_ of 15 µmol/L, and ventricular cells were largely protected against this effect [43]. In contrast to this, increased peak I_Na_ and I_K1_ were reported in hiPSC-CMs following 24-h pre-incubation with 1 µmol/L empagliflozin [14]. We also found here (Figs. 3 and 5) that chronic in vivo empagliflozin treatment in *db/db* + Aldo restored I_K1_, RMP, and dV/dt_max_, a surrogate of restored peak I_Na_ function. In vivo empagliflozin treatment also rescued other K^+^ currents (I_to_, I_K,slow_, and I_sus_) that were downregulated in *db/db* + Aldo (Fig. 5D). Notably, these electrophysiological changes were observed in isolated myocytes, where residual empagliflozin should be largely washed out during the isolation and pre-incubation conditions. Thus, we attribute these effects to be longer-lasting effects of empagliflozin on either the channels or the cellular milieu (e.g., ROS or CaMKII activation state).

In murine HFrEF ventricular myocytes, acute empagliflozin (10 µmol/L) reversed I_Na,L_ enhancement (with minimal effect on peak I_Na_) [46], and empagliflozin inhibited the H_2_O_2_-induced I_Na,L_ with an IC_50_ of 0.79 µmol/L in HEK293T cells expressing human Na_V_1.5 channels [46] and with an IC_50_ of 5.7 µmol/L in adult murine ventricular myocytes [24]. Interestingly, 1 µmol/L empagliflozin acutely did not inhibit I_Na,L_ in the HFD + L-NAME HFpEF mouse model; however, a 4-h cell pretreatment with empagliflozin fully reversed I_Na,L_ upregulation in HFpEF myocytes, and this mimicked the effect of selective CaMKII inhibition [24]. Here, we report that chronic in vivo empagliflozin treatment in *db/db* + Aldo mice reverses elevated I_Na,L_ in ventricular myocytes, even in the absence of empagliflozin during recordings, indicating functional restoration rather than acute inhibition of I_Na,L_ (Fig. 6). Maximum plasma concentrations (C_max_) in patients receiving 10 mg/day and 25 mg/day empagliflozin were 0.23–0.37 µmol/L and 0.50–0.73 µmol/L, respectively [52]. Thus, supratherapeutic (3–30 µmol/L) empagliflozin concentrations may directly inhibit Na^+^ channels; however, empagliflozin reverses the regulatory functional changes of Na^+^ channels via indirect mechanisms already at clinically relevant (≤ 1 µmol/L) concentrations.

### Pleiotropic effects of empagliflozin on cardiomyocyte signaling and gene expression

Empagliflozin likely has pleiotropic effects; thus, multiple mechanisms could contribute to reduced arrhythmia susceptibility, including attenuation of hyperglycemia, diastolic dysfunction, mechanical afterload, post-translational regulatory modifications, and changes in expression of key ion channels and Ca^2+^ handling proteins [12, 15, 35]. Although empagliflozin did not result in complete reversion of the transcriptome to control, we did observe reversals in key signaling pathways, including Nos1, PKG, PI3K–Akt, and metabolic and profibrotic genes that could partially explain some of the electrophysiological changes observed in our functional experiments (Fig. 8E, S2, and S3). Diabetic hyperglycemia has also been shown to regulate multiple ionic currents and ECC mechanisms mediated by increased CaMKII *O*-GlcNAcylation and ROS-mediated PKC activation [20–22]. Here, we report that in vivo empagliflozin treatment in *db/db* + Aldo mice downregulates genes related to PKC activation (Fig. 9D). Empagliflozin was shown to improve diastolic function in diabetic patients with or without HFpEF [48], and in multiple HFpEF models, including obese-diabetic Zucker fatty and spontaneously hypertensive ZSF1 rats [51], deoxycorticosterone acetate salt-sensitive rats [11], and *db/db* + Aldo mice here (Fig. 2). In human HFpEF fibers, empagliflozin reduced myofilament passive stiffness by enhancing phosphorylation levels of regulatory myofilament proteins (titin, troponin-I, myosin binding protein-C) [44]. In line with this, we previously reported key changes in phosphorylation of myofilament proteins in *db/db* + Aldo that were sex-dependent with higher titin PEVK spring element phosphorylation and reduced troponin-I phosphorylation in female mice [36].

Increased mechanical afterload prolongs APD_90_ and alters ionic currents via nitric oxide (NO) signaling [25], and empagliflozin was shown to inhibit a Na^+^/H^+^ exchanger 1 (NHE1)–NO pathway [9]. Here, empagliflozin treatment in *db/db* + Aldo mice restored NOS1 expression (Fig. 8E) and reversed the upregulation of genes associated with ROS (Fig. S5). Empagliflozin also reduced the level of total *O*-GlcNAc-modified proteins, CaMKII autophosphorylation, and phosphorylation of RyR2 at serine 2814 (a CaMKII target site), and attenuated Ca^2+^ sparks and waves in *db/db* and TAC mice and failing human cardiomyocytes [29, 39], indicating an additional mechanism by which SGLT2 inhibitors could provide benefits in HFpEF [18]. Here, in vivo empagliflozin treatment also ameliorated the slowing of CaT decay tau and the pacing-induced elevation of diastolic [Ca^2+^]_i_ in male *db/db* + Aldo ventricular myocytes (Table S2), indicating improved SR Ca^2+^ reuptake, in line with increased phosphorylation of PLN (Fig. 10). In addition to this, empagliflozin also rescued the I_Ca,L_ reduction in *db/db* + Aldo (Fig. 6A).

Empagliflozin may also exert additional cardioprotective effects through actions on non-cardiomyocytes in the heart, including improved NO signaling and vascular tone associated with reduced inflammation and ROS production in coronary endothelial cells [60] and attenuation of endothelial-to-mesenchymal transition and fibroblast activation [53]. HFpEF is characterized by pronounced coronary microvascular dysfunction, although the underlying molecular mechanisms remain incompletely understood [26]. Reduced coronary blood flow impairs myocardial oxygen delivery, thereby compromising cardiomyocyte metabolism and function in the failing heart [33]. In a non-diabetic mouse model of acute myocardial infarction, empagliflozin reduced infarct size, improved microvascular function, and largely reversed ischemia–reperfusion-induced transcriptomic alterations, predominantly in endothelial cells, via Stat3 (Signal transducer and activator of transcription 3) signaling [27, 41]. Collectively, these effects may limit cardiac fibrosis, enhance coronary perfusion, and confer beneficial paracrine effects on cardiomyocytes, thereby further reducing arrhythmia susceptibility in HFpEF.

Gene expression analysis identified differentially expressed gene clusters specific to *db/db* genotype, aldosterone infusion, and empagliflozin treatment (Figs. 8 and 9). We have previously shown that *db/db* mice (without aldosterone) and aldosterone-treated WT mice only exhibited mild diastolic dysfunction without pulmonary congestion and HF symptoms, and that the combination of *db/db* and excess aldosterone synergistically promoted cardiac hypertrophy, BNP elevation, and severe diastolic dysfunction with pulmonary congestion [23]. The predominantly *db/db*-dependent and aldosterone-dependent gene clusters provide mechanistic insights into their differential individual contributions to gene expression changes in *db/db* + Aldo (Fig. 9). Importantly, empagliflozin changed the overall gene expression profile in *db/db* + Aldo, but instead of moving the altered gene expression toward control (WT + Veh), empagliflozin shifted it further to a new expression profile (Fig. 8). Nonetheless, empagliflozin rescued expression of some genes related to lipid and protein metabolism, inflammation, Notch signaling, phosphoinositide-3-kinase (PI3K)–Akt, PKC, PPAR and SUMO pathways, ubiquitin and ER-stress in *db/db* + Aldo hearts. Recent proteomic data in the HFD + L-NAME HFpEF mouse model revealed attenuation of cardiac inflammation via inhibition of Stat1 (Signal transducer and activator of transcription 1), an interferon response transcription factor following 4-week empagliflozin treatment [55]. In this study, empagliflozin also reduced the expression of Stat1 and interferon beta and interleukin-1 signaling genes in *db/db* + Aldo hearts (Fig. 9D, Online Resource 1–2). However, there were no systematic gene expression changes that closely correlate with most of the electrophysiological or Ca^2+^ handling alterations except for SERCA2 expression changes. These data suggest that the significant functional improvements by empagliflozin in *db/db* + Aldo are likely to involve key regulatory post-translational modifications, protein stability/recycling, and a reduced inflammatory response. Inhibition of histone deacetylase 6 (HDAC6) was also shown to restore gene expression related to hypertrophy, fibrosis, and mitochondrial energy production in HFD + L-NAME HFpEF hearts, and HDAC6 inhibition may have synergistic effects with empagliflozin treatment [49]. This is in line with our data that cardiomyocyte hypertrophy and gene expression profiles were not restored by empagliflozin, and it also provides new directions for further improving treatments for patients with cardiometabolic HFpEF.

## Conclusions, limitations, and perspectives

We found that 4-week oral treatment with the SGLT2 inhibitor empagliflozin rescued the functional HFpEF phenotype in *db/db* + Aldo mice. In empagliflozin-treated HFpEF mice, ventricular cardiomyocyte electrophysiology, Ca^2+^ handling, and proarrhythmia mechanisms were all reversed even in the absence of empagliflozin during the recordings, indicating a true phenotypic rescue and functional ECC reverse remodeling. However, heart and cardiomyocyte hypertrophy were not reversed by empagliflozin. Moreover, only a few gene expression changes were reversed in empagliflozin-treated *db/db* + Aldo murine hearts, and the overall gene expression profile was shifted further away from control. As more human HFpEF heart samples become available, including those who have been treated with empagliflozin, future studies are needed to assess empagliflozin’s effect on cardiac transcriptional and proteomic remodeling and post-translational regulation in the different cell types in the heart [5, 16, 41]. The exact antiarrhythmic benefits of empagliflozin in HFpEF require further studies both in human patients and in vivo animal models.

## Supplementary Information

Below is the link to the electronic supplementary material.

Supplementary file1 (PDF 2753 KB)

## Data Availability

The data underlying this article have been made publicly available at Dryad and can be accessed at 10.5061/dryad.prr4xgz2g. Sequencing data have been deposited in GEO (accession number: GSE285966).
