# Supplementary material for: Empagliflozin restores cardiac function in obese-diabetic HFpEF mice but further alters gene expression
Source: Basic Res Cardiol. 2026 May 21;121(4):669–88. doi: 10.1007/s00395-026-01184-7 (PMC13372850; doi:10.1007/s00395-026-01184-7)
Supplement: Supplementary file 1 — Supplementary file1 (PDF 2753 KB) [file 395_2026_1184_MOESM1_ESM.pdf]

SUPPLEMENTARY INFORMATION

Empagliflozin restores cardiac function in obese-diabetic HFpEF mice but further alters gene expression

Juliana Mira Hernandez,[1,2,*] Logan R.J. Bailey,[3,4,*] Victoria R. Salemme,[1] Christopher Y. Ko,[1] Erin Y. Shen,[1] Megan Ngim,[1] Duong T. Hoang,[1] Melissa Corea,[1] Julie Bossuyt,[1] Jennifer M. Davis,[3,4] Donald M. Bers,[1] Bence Hegyi[1]

[1]Department of Pharmacology, University of California, Davis, CA, USA
[2]Research Group Biogenesis, Faculty of Agricultural Sciences, Veterinary Medicine, University of Antioquia, Medellin, Colombia
[3]Institute for Stem Cell and Regenerative Medicine, University of Washington, Seattle, WA, USA
[4]Department of Lab Medicine and Pathology, University of Washington, Seattle, WA, USA

*equal contributions

Correspondence:
Hegyi Bence, MD, PhD
Department of Pharmacology
University of California, Davis
451 Health Sciences Drive
Davis, CA 95616, USA
Phone: 530-752-9962
E-mail: bhegyi@ucdavis.edu

AND

Donald M. Bers, PhD
Department of Pharmacology
University of California, Davis
451 Health Sciences Drive
Davis, CA 95616, USA
Phone: 530-752-6517
Email: dmbers@ucdavis.edu

SUPPLEMENTAL METHODS

Implantation of osmotic minipumps

Osmotic minipumps (Alzet, model 2004) were prepared per manufacturer's instructions. Animals were anesthetized by isoflurane (1-5%) during the entire surgical procedure. Once the animal was anesthetized, fur on the back was removed with a depilatory. The operating field was disinfected with chlorhexidine solution and 70% ethanol. A 1.5 cm mid-scapular incision was made across the back perpendicular to the spine. A haemostatic clamp was inserted into the incision, and the subcutaneous tissue was spread by opening and closing the jaws of the haemostat to create a pocket for the pump. A pump filled with 200 µL solution was inserted into the pocket, starting with the delivery portal. The wound was closed with pharmaceutical grade surgical glue. Additionally, for any incisions greater than 1.5 cm or when glue did not provide sufficient closure, 3 single non-continuous sutures were placed and removed 7-10 days later. Recovery of animals was carefully monitored, and a dose of analgesic (buprenorphine, 0.05-0.1 mg/kg, subcutaneous) was given during surgery, and additional doses were given if pain and distress persisted upon re-evaluation every 12 hr over a 48-hr period. The pumps stayed in the mice for 4 weeks and continuously delivered *d*-aldosterone (0.3 µg/hour) or vehicle (5% ethanol in saline).

BNP measurement

B-type natriuretic peptide (BNP) levels were measured from blood plasma using enzyme-linked immunosorbent assay (ELISA) kits specific to mouse BNP (RayBiotech, EIAM-BNP-1) according to the manufacturers' instructions. Plasma was prepared from anticoagulated (EDTA) blood by centrifugation at 3220 g for 1 hour at 4°C. The protein concentrations were calculated using a standard curve generated with recombinant standards provided by the manufacturers. Three technical replicates were performed for each biological sample.

Murine echocardiography

Systolic and diastolic ventricular heart functions of mice were assessed by transthoracic echocardiography using the Vevo 2100 echocardiography system (FUJIFILM VisualSonics, Toronto, ON, Canada) equipped with a 40 MHz linear probe. Mice fur was removed by a depilatory the day before echocardiography recordings. During recordings, mice were anesthetized with isoflurane inhalation (1.5%), which was later individually adjusted (between 1 to 3%) to achieve a stable heart rate between 350 to 450 beats/min to assess diastolic dysfunction (to avoid fusion of the waves) and 450 to 600 beats/min to assess systolic cardiac function (to avoid depressed contractile function) in each animal. ECG monitoring was obtained using limb electrodes, and core temperature was carefully monitored and maintained at 37ºC during the entire procedure. Left ventricular (LV) M-mode echocardiography in parasternal short-axis view was performed for assessment of LV dimensions and systolic function. Pulsed wave Doppler and tissue Doppler images were acquired to assess diastolic function. At least three consecutive cardiac cycles were sampled for each measurement taken, and blinded analysis was performed off-line.

Enzymatic isolation of ventricular cardiomyocytes

Mice injected with heparin (400 U/kg body weight) and anesthetized with isoflurane (5% in an induction chamber, then 1.5-3% via nose cone). Deep surgical anaesthesia was confirmed by abolished pain reflexes. All animals were euthanized by surgical excision of the heart while in deep anaesthesia. Hearts were excised and rinsed in cold nominally Ca^{2+}-free Minimal Essential Medium. The aorta was cannulated and retrograde perfused on constant flow Langendorff apparatus at 37ºC with Ca^{2+}-free normal Tyrode's solution, gassed with 100% O_2. Then, the heart was perfused for 10-15 min with 100 mg collagenase (type 2, Worthington Biochemical Corp., Lakewood, NJ, USA) and 1.4 mg protease (type XIV, Sigma-Aldrich, St. Louis, MO, USA) in 50 mL Tyrode's solution (with 10 µmol/L Ca^{2+}) to enzymatically isolate cardiomyocytes predominantly from the midmyocardial region of the left ventricular (LV) free wall. Following digestion, the myocytes were gently triturated with a pipette, then filtered through a nylon mesh and allowed to sediment for ~10 min. The sedimentation was repeated three times using increasing [Ca^{2+}] from 0.125 to 0.25 then 0.5 mmol/L. Finally, ventricular myocytes were kept in Tyrode's solution (0.5 mmol/L [Ca^{2+}]) at room temperature until use.

Calcium imaging

Intracellular [Ca^{2+}] transients and diastolic Ca^{2+} events (sparks) were measured in freshly isolated ventricular cardiomyocytes loaded with Fluo-4 AM (10 µmol/L, Invitrogen, Waltham, MA, USA) and Pluronic F-127 (0.02%, Invitrogen). The dye was loaded for 30 minutes at room temperature followed by wash and de-esterification for 30 minutes. Fluo-4 was excited at 488 nm using an Argon laser, and emission was collected using a 500-530 nm bandpass filter. Images were recorded using confocal microscopy in line scan mode (Bio-Rad Radiance 2100, Hercules, CA, USA) using a 40x objective and scanned at 6 ms/line. Intact cardiomyocytes were plated on laminin-coated coverslips and paced at 1 Hz and 2 Hz for 30 beats at each pacing rate in a field stimulation chamber (Warner Instruments, Hamden, CT, USA). Myocytes were continuously perfused with Tyrode's solution containing (in mmol/L): NaCl 140, KCl 4, $CaCl_2$ 1.8, $MgCl_2$ 1, HEPES 5, Na-HEPES 5, glucose 5.5; pH=7.40. After pacing was stopped, Ca^{2+} sparks were recorded for 1 min. Sarcoplasmic Ca^{2+} content was then assessed by local delivery of 10 mmol/L caffeine. During analysis, the non-cellular background fluorescence was subtracted from all measured fluorescence, and the initial baseline fluorescence (F_0) in myocytes quiescent for long time periods was determined in each cell. There was no statistical difference in F_0 values at rest between any experimental groups. Diastolic [Ca^{2+}] variations (during pacing) were assessed by measurements of minimum diastolic Ca^{2+} fluorescence during pacing and the ratio of diastolic/resting fluorescence after background subtraction (F/F_0). Only recordings that showed no spontaneous Ca^{2+} waves or spontaneous CaT were included in the Ca^{2+} spark analysis. ImageJ was used for image processing and analysis, and Ca^{2+} sparks were analysed using the SparkMaster plugin.

Cellular electrophysiology

Isolated single left ventricular murine cardiomyocytes were placed in a temperature-controlled perfusion chamber (Warner Instruments) mounted on a Leica DMI3000 B inverted microscope (Leica Microsystems, Buffalo Grove, IL, USA). Cells were bathed at 37°C (for 10 minutes before starting the experiments) and continuously perfused (2 mL/min) with Tyrode's solution. Electrodes were fabricated from borosilicate glass (World Precision Instruments., Sarasota, FL, USA) having tip resistances of 2 to 2.5 MΩ when filled with an internal solution. Axopatch 200B amplifier (Axon Instruments Inc., Union City, CA, USA) was used for recordings, and the signals were digitized at 50 kHz by a Digidata 1322A A/D converter (Axon Instruments) under software control (pClamp10.4). Series resistance was typically 3 to 5 MΩ, and it was compensated by ≥90%. Experiments were discarded when the series resistance was high or increased by ≥20% during the recordings. The osmolality of all applied solutions was adjusted to 290-300 mOsm/L using a vapor pressure osmometer (Vapro 5520; Wescor Inc., Logan, UT, USA). The pH of all applied solutions was regularly checked and carefully adjusted using a pH meter (VWR sympHony SB70P; VWR, Radnor, PA, USA). All electrophysiology experiments were conducted at 37±0.2°C.

APs were recorded in whole-cell I-clamp conditions where cells were stimulated using supra-threshold depolarizing pulses (2 ms duration) delivered via the patch pipette at 1 Hz frequency. The Tyrode's solution for bath perfusion contained (in mmol/L): NaCl 140, KCl 4, $CaCl_2$ 1.8, $MgCl_2$ 1, HEPES 5, Na-HEPES 5, glucose 5.5; pH=7.40. Pipette solution contained (in mmol/L): K-aspartate 100, KCl 30, NaCl 8, Mg-ATP 5, phosphocreatine dipotassium salt 10, HEPES 10, EGTA 0.01, cAMP 0.002, and calmodulin 0.0001; pH=7.20 (with KOH). Using this solution, the intracellular Ca^{2+} transient and contraction of the cardiomyocyte were preserved. AP durations at 20, 50, 75, and 90% repolarization (APD_{20}, APD_{50}, APD_{75}, and APD_{90}, respectively) were used to characterize AP repolarization dynamics. Series of 50 consecutive APs were analysed to estimate short-term variability (STV) of APD_{90} according to the following formula: $STV=\Sigma(|APD_{n+1}-APD_n|)/[(n_{beats}-1)\times\sqrt{2}]$, where APD_n and APD_{n+1} indicate the durations of the nth and $(n+1)^{th}$ APs, and n_{beats} denotes the total number of consecutive beats analysed. Diastolic arrhythmogenic activities were elicited by cessation of 1-min burst pacing (10 Hz), and membrane potential was recorded for an additional 1 minute. Delayed afterdepolarizations (DADs) were defined as an increase in resting membrane potential exceeding 1 mV in amplitude within 1 second.

K^+ currents were recorded in whole-cell V-clamp experiments using an internal solution containing (in mmol/L): K-Aspartate 100, KCl 20, NaCl 8, Mg-ATP 5, EGTA 10, $CaCl_2$ 4.1, HEPES 10, cAMP 0.002,

phosphocreatine-K_2 10, and calmodulin 0.0001, with pH=7.2 (free $[Ca^{2+}]_i$=100 nmol/L, calculated using the WEBMAXC Extended version of the MaxChelator software, https://somapp.ucdmc.ucdavis.edu/pharmacology/bers/maxchelator/webmaxc/webmaxcE.htm), and in the presence of Na^+ and Ca^{2+} current inhibitors, including tetrodotoxin (TTX, 10 µmol/L, Alomone Labs, Cat#: T-550) for I_{Na}, and nifedipine (10 µmol/L, Sigma, Cat#: N7634) for I_{Ca} in the perfusing Tyrode's solution. Different K^+ current components were separated using biexponential fitting (R^2>0.9 in each case) to the decay of the voltage-gated outward K^+ currents (I_{Kv}). Voltage-gated K^+ currents were elicited using a 4.5 s-long test pulse to +60 mV from a holding potential of –80 mV with an interpulse interval of 5.5 s. I_{K1} current traces were analysed at the end of 500-ms test pulses to –140 mV (inward I_{K1}) and –40 mV (outward I_{K1}) from the holding potential of −80 mV.

$I_{Na,L}$ was recorded using an internal solution containing (in mmol/L): CsCl 110, tetraethylammonium chloride 20, Mg-ATP 5, HEPES 10, phosphocreatine disodium salt 5, calmodulin 0.0001, EGTA 10, $CaCl_2$ 4.1 (free $[Ca^{2+}]$=100 nmol/L), pH=7.20. Bath solution contained (in mmol/L): NaCl 140, CsCl 4, $CaCl_2$ 1.8, $MgCl_2$ 1, HEPES 5, Na-HEPES 5, glucose 5.5, 4-aminopyridine 5, nifedipine 0.01, pH=7.40. $I_{Na,L}$ was measured at the end of a 500 ms depolarizing pulse to –40 mV from a –120 mV holding potential (to maximize Na^+ channel availability). $I_{Na,L}$ could be inhibited by TTX (10 µmol/L), and the TTX-sensitive current amplitude was reported in each cell.

$I_{Ca,L}$ was recorded using the same internal solution as listed above for $I_{Na,L}$ measurements. The bath solution was similar to that used for $I_{Na,L}$ measurements, but nifedipine was replaced with TTX (10 µmol/L). I_{CaL} was measured using 500 ms-long voltage steps to test potentials between –40 mV and +20 mV from a holding potential of –80 mV every 5 s with a 50 ms pre-step to –40 mV to inactivate Na^+ channels. At the end of each experiment, I_{CaL} was inhibited using nifedipine (10 µmol/L).

All ionic currents were normalized to cell capacitance (i.e., current density), determined in each cell using short (10 ms) hyperpolarizing pulses from −10 mV to −20 mV.

Chemicals and reagents were purchased from Sigma-Aldrich (St. Louis, MO, USA), if not specified otherwise. Empagliflozin was from MedChemExpress (Cat. No.: HY-15409, Monmouth Junction, NJ, USA).

Data processing, analysis, and plotting have been performed using Clampfit 10 (Molecular Devices, San Jose, CA, USA), Excel for Office 365 (Microsoft, Redmond, WA, USA), GraphPad Prism 10 (GraphPad Software, Boston, MA, USA) and Origin 2016 (OriginLab, Northampton, MA, USA) software.

RNA-sequencing and analysis

Total RNA was extracted from whole-heart lysates using TRIzol and the Qiagen RNeasy kit. RNA-sequencing was performed by BGI Genomics using paired-end 100 nucleotide sequencing with 40 million reads. Cleaned reads were aligned to reference genomes (mouse: GRCm38/mm10) using STAR [1] with *de novo* junction discovery and summarized at the gene level using featureCounts [3]. Differential expression analysis was completed using DESeq2 using the standard workflow [4]. Batch correction for different sequencing runs between HFpEF models was performed using limma's removeBatchEffect function using the standard workflow [5]. Pathway and ontology enrichment analyses for all experiments were performed using gprofiler2 [2]. Heatmaps were generated using the pheatmap package in R. To generate gene-gene correlation data, gene-gene Spearman correlations were calculated for all genes that were differentially expressed between *db/db*+Aldo and WT+Vehicle control animals. K-nearest neighbor (KNN) clustering was subsequently performed on the Spearman correlations using 5 clusters as this was the number of large clusters revealed by hierarchical clustering and clustering with more than 5 clusters began to produce small clusters with few constituents. To compare cluster functional similarity, pairwise Jaccard similarity indices were calculated by dividing the intersection of enriched GO:BP terms between two clusters by the union of all GO:BP terms enriched between the two clusters. Normalized expression values for genes and cluster assignments for each gene are provided in Supplemental Datasets (Online Resource 1-7). Sequencing data have been deposited in GEO (accession number: GSE285966).

Protein analysis

Ventricular tissues from *db/db*+Aldo HFpEF and healthy WT+Vehicle control mice with and without chronic *in vivo* empagliflozin treatment were collected and rinsed three times in ice-cold nominally Ca^{2+}-free normal Tyrode's solution, then cut in 6-12 pieces, flash-frozen, and stored at −80°C until use.

Hearts were homogenized in ice-cold buffer containing (in mmol/L): NaCl 300, Tris-HCl (pH=7.4) 40, NaF 20, tetrasodium pyrophosphate 2, $MgCl_2$ 1, EGTA 2, EDTA 2, 4% NP40, and protease and phosphatase inhibitors (EMD Millipore, set III and V, respectively). Protein concentration was assessed in heart homogenates with a Pierce BCA protein assay (Thermo Fisher Scientific, Cat# 23225). Proteins were divided using Tris-HCl SDS-PAGE electrophoresis gels (4–20% Criterion TXT Bio-Rad) and then transferred to a 0.22 µm PVDF membrane that was then blocked with 3% BSA in TBST (Sigma Aldrich) for phosphorylated proteins or 5% blotting-grade blocker (Bio-Rad) for total proteins. Protein transfer success was checked with Ponceau staining. Blots were incubated overnight at 4°C with primary antibodies: SERCA2 (1:500, mouse, ThermoFisher, Cat# MA3-919), PLN (1:5000, ThermoFisher; mouse, Cat# MA3-922), pS16-PLN (1:1000, rabbit, Badrilla, Cat# A010-12AP), pT17-PLN (1:1000, rabbit, Badrilla, Cat# A010-13AP), CaMKIIδ (1:1000, rabbit, ThermoFisher, Cat# PA5-22168), pT287-CaMKII (1:250, rabbit, PhosphoSolutions, Cat# p1005-286), and GAPDH (1:1000, mouse; Bio-Rad, Cat# MCA4739). After primary antibody incubation, membranes were rinsed with TBS-Tween (TBST) and then incubated at room temperature with secondary antibodies for 1 hour: Alexa Fluor 647 (1:10000, goat anti-rabbit; Invitrogen, Cat# A32733) and Alexa Fluor 790 (1:10000, goat anti-mouse, Invitrogen, Cat# A11357). Later, the blots were rinsed in TBST and then left washing in more TBST for 30 min until reading. Membranes were stripped using Restore PLUS Westen Blot Stripping Buffer (ThermoScientific, Cat# 46430) for 2 to 5 minutes before re-blocking and applying a different primary antibody. Blots were imaged in the ChemiDoc MP Imaging System (Bio-Rad) and analysed with Image Lab (Bio-Rad). For molecular weight markers, Precision Plus Protein Dual Color Standards (Bio-Rad) were used. Two technical replicates were prepared for each blot.

Statistical analysis

Pooled data are presented as Mean ± SD. Normality of the data was assessed by Shapiro-Wilk test and the equality of group variance was tested using Brown-Forsythe test. Statistical significance of differences was determined using ANOVA and Kruskal-Wallis test when data were not normally distributed, followed by appropriate multiple comparisons tests. Pairwise comparisons were made between the following groups (prespecified in experimental design): (1) WT+Vehicle without Empa *vs.* WT+Vehicle with Empa (2) WT+Vehicle without Empa *vs. db/db*+Aldo without Empa, (3) *db/db*+Aldo without Empa *vs. db/db*+Aldo with Empa, (4) WT+Vehicle with Empa *vs. db/db*+Aldo with Empa. Multiplicity adjusted *P* values were reported for each comparison. GraphPad Prism 11 (Boston, MA, USA) software was used for statistical analysis. Blinded data acquisition and analysis have been performed for all *in vivo* measurements. Animals were grouped with no blinding but randomized in cellular experiments. Fully blinded analysis was not performed in cellular studies because the same person carried out the experiments and analysis. For proper allocation concealment, animals were recruited blinded based on sequential ear tag numbers randomly assigned by the animal housing staff. Male and female animals were used in equal numbers. Group sizes were determined by an *a priori* power analysis for a two-tailed *t*-test with an α of 0.05 and power of 0.8, in order to detect a 20% difference signal at the endpoint. Representative traces/images reflected the average behaviour in each experiment.

Illustrations in Figure 1 were generated in part using Servier Medical Art, provided by Servier, licensed under a Creative Commons Attribution 3.0 unported license.

	**WT+Vehicle w/o Empa**		**WT+Vehicle w/ Empa**		***db/db*+Aldo w/o Empa**		***db/db*+Aldo w/ Empa**	
Sex	Male	Female	Male	Female	Male	Female	Male	Female
N (animals)	8	8	8	8	7	8	8	8
Body weight (g)	30.4±1.6	22.9±1.4 $^{P<0.001}$	28.5±2.8	25.9±2.6 $^{P=0.07}$	54.1±4.8	57.4±3.3	53.6±6.3	53.4±7.2
Blood glucose (mmol/L)	11.3±1.7	10.4±1.8	11.6±2.0	11.0±1.6	22.7±7.1	23.3±8.9	14.2±4.6	12.4±5.5
BNP (pg/mL)	56.6±12.3	68.9±14.3	43.8±5.6	46.6±16.0	310.5±32.5	210.6±58.3 $^{P=0.02}$	62.9±24.5	55.2±4.0
Lung (wet/dry weight)	4.18±0.20	4.22±0.24	4.27±0.14	4.36±0.14	4.75±0.63	4.60±0.37	4.21±0.10	4.14±0.13
HW / TL (g/mm)	1.01±0.08	0.83±0.07 $^{P<0.001}$	0.93±0.12	0.85±0.06 $^{P=0.10}$	1.06±0.04	1.02±0.12	1.10±0.09	1.05±0.10
Liver (g)	1.39±0.12	1.07±0.09 $^{P<0.001}$	1.25±0.21	1.22±0.09	3.89±0.74	3.63±0.58	3.24±0.70	3.01±0.62
Kidney (g)	0.36±0.02	0.28±0.03 $^{P=0.005}$	0.36±0.04	0.34±0.04	0.50±0.03	0.46±0.06	0.64±0.02	0.57±0.09
Gastroc+Sol muscles (g)	0.32±0.03	0.25±0.01 $^{P=0.005}$	0.28±0.04	0.28±0.04	0.19±0.01	0.17±0.01	0.22±0.03	0.22±0.03
Ejection fraction (%)	78.7±1.5	79.0±1.6	78.4±4.5	79.4±2.5	80.5±1.7	80.2±1.6	80.0±2.0	79.4±2.4
Fractional shortening (%)	46.3±1.4	46.5±1.6	46.2±4.3	47.1±2.6	48.0±1.9	47.7±1.5	47.9±1.9	47.2±2.2
LVIDd (mm)	3.42±0.24	3.32±0.19	3.47±0.31	3.42±0.25	3.29±0.32	3.29±0.32	3.77±0.26	3.67±0.48
LVPWd (mm)	0.86±0.04	0.81±0.06 $^{P=0.07}$	0.80±0.07	0.78±0.05	0.98±0.07	0.98±0.05	0.94±0.02	0.94±0.06
LV Mass (g)	103.1±11.3	88.9±11.3 $^{P=0.03}$	99.1±13.6	92.5±5.6	124.7±20.8	127.7±14.6	130.7±9.4	124.0±22.3
E / A	1.34±0.15	1.39±0.21	1.46±0.18	1.42±0.24	1.34±0.27	1.75±0.56 $^{P=0.10}$	1.48±0.23	1.55±0.23
E / e’	27.5±2.9	29.5±0.9	26.7±2.3	25.5±2.0	42.7±5.6	49.6±7.5 $^{P=0.07}$	34.3±4.6	34.6±3.6
LA area (mm^2)	2.29±0.05	2.21±0.04 $^{P=0.003}$	2.28±0.07	2.25±0.05	2.95±0.15	3.03±0.17	2.63±0.10	2.60±0.08

Table S1. Sex-dependent differences in morphometric data and echocardiographic parameters in empagliflozin-treated HFpEF mice. Male-female comparisons were made in each experimental group. Male-female differences were determined in each group using unpaired *t*-test, and *P* values are reported (if $P \leq 0.10$) after each value in females.
(WT, wild-type; Empa, empagliflozin; Aldo, aldosterone; BNP, B-type natriuretic peptide; HW/TL, heart weight to tibial length ratio; gastroc+sol, gastrocnemius and soleus muscles; LVIDd, left ventricular internal diameter at diastole; LVPWd, left ventricular posterior wall thickness at diastole; E/A, mitral E wave to A wave ratio; E/e’, mitral E wave to e’ wave ratio; LA area, left atrial area)

	**WT+Vehicle w/o Empa**		**WT+Vehicle w/ Empa**		***db/db*+Aldo w/o Empa**		***db/db*+Aldo w/ Empa**	
Sex	Male	Female	Male	Female	Male	Female	Male	Female
N (animals)	4	4	4	4	4	4	4	4
n (cells)	26	28	37	36	27	31	37	33
C_m (pF)	151±12	145±9 $^{P=0.051}$	150±5	152±7	170±12	168±14	169±8	170±11
APD_{90} @ 1 Hz (ms)	50.0±8.6	48.1±5.4	40.3±4.1	40.3±2.9	74.9±7.7	78.8±14.3	50.9±5.1	52.8±4.1
STV @ 1 Hz (ms)	0.73±0.23	0.83±0.21	0.84±0.19	0.76±0.23	2.07±0.52	1.48±0.50 $^{P=0.007}$	0.93±0.26	1.02±0.18
APD_{90} alternans (ms)	0.11±0.06	0.33±0.36	0.43±0.59	0.29±0.21	13.0±8.9	15.3±17.3	4.9±7.2	3.5±8.0
I_{K1} @ -140 mV (A/F)	-26.9±4.0	-26.0±3.3	-27.1±5.6	-24.5±3.0	-18.7±2.4	-16.5±2.2 $^{P=0.02}$	-24.6±2.8	-25.2±3.5
I_{K1} @ -40 mV (A/F)	1.81±0.45	1.64±0.52	1.81±0.50	1.65±0.46	1.31±0.29	1.36±0.35	1.73±0.37	1.81±0.37
I_{to} @ +60 mV (A/F)	18.2±8.3	17.7±6.9	21.0±8.8	20.1±6.6	9.4±3.0	9.4±2.8	17.6±8.3	14.8±4.4
$I_{K,slow}$ @ +60 mV (A/F)	7.8±4.9	8.1±2.6	6.7±2.8	8.2±3.3	5.4±2.8	5.3±3.0	6.4±2.0	6.9±2.5
I_{sus} @ +60 mV (A/F)	6.3±2.4	4.9±2.0	5.3±1.2	5.3±1.9	3.6±1.4	4.6±2.1	7.2±2.0	5.7±2.3
$I_{Na,L}$ @ -40 mV (A/F)	-0.42±0.07	-0.40±0.06	-0.40±0.07	-0.39±0.09	-0.92±0.09	-0.78±0.09 $^{P=0.01}$	-0.44±0.07	-0.41±0.06
$I_{Ca,L}$ @ 0 mV (pA/pF)	-9.7±2.5	-8.3±1.8	-9.3±1.2	-9.3±2.3	-6.7±1.1	-8.0±2.6	-9.0±1.4	-10.0±1.5
Diastolic $[Ca^{2+}]$ (F/F_0)	1.57±0.33	1.64±0.41	1.78±0.27	1.71±0.23	2.18±0.47	1.77±0.38 $^{P=0.075}$	1.76±0.25	1.67±0.50
Peak $[Ca^{2+}]$ (F/F_0)	5.6±1.8	5.2±1.4	5.2±1.2	4.8±1.9	5.5±2.2	4.9±2.0	5.7±2.1	5.2±1.3
CaT amplitude ($\Delta F/F_0$)	4.0±1.6	3.5±1.1	3.4±1.1	3.1±1.9	3.3±1.8	3.2±1.7	4.0±2.0	3.3±0.6
CaT decay τ (ms)	385±127	426±198	420±74	402±95	582±98	408±82 $^{P=0.005}$	388±98	422±78
SR Ca^{2+} load ($\Delta F/F_0$)	7.6±2.2	6.0±1.9	6.9±2.5	6.4±2.2	7.7±2.7	8.6±2.9	7.1±3.3	7.7±3.1

Table S2. Sex-differences in electrophysiological and Ca^{2+} handling parameters in empagliflozin-treated HFpEF mice.
Male-female comparisons were made in each experimental group. Male-female differences were determined in each group using unpaired *t*-test and Mann-Whitney *U*-test. *P* values are reported (if $P \leq 0.10$) after each value in females.
(WT, wild-type; Empa, empagliflozin; Aldo, aldosterone; C_m, membrane capacitance; APD_{90}, action potential duration at 90% of repolarization; STV, short-term variability of APD_{90}; I_{K1}; inward rectifier K^+ current; I_{to}, transient outward K^+ current; $I_{K,slow}$, slowly inactivating K^+ current; I_{sus}, sustained current; $I_{Na,L}$, late Na^+ current; $I_{Ca,L}$, L-type Ca^{2+} current; CaT, intracellular $[Ca^{2+}]$ transient; SR, sarcoplasmic reticulum)

	**WT+Vehicle w/o Empa**		**WT+Vehicle w/ Empa**		***db/db*+Aldo w/o Empa**		***db/db*+Aldo w/ Empa**	
Sex	Male	Female	Male	Female	Male	Female	Male	Female
N (animals)	4	4	4	4	4	4	4	4
SERCA2/GAPDH (norm.)	0.92±0.06	1.08±0.18	0.92±0.03	0.91±0.13	0.71±0.09	0.76±0.06	0.74±0.05	0.89±0.10 $^{P=0.057}$
CaMKII/GAPDH (norm.)	0.92±0.03	1.08±0.17	0.76±0.23	0.91±0.13	0.84±0.08	0.94±0.33	0.86±0.18	1.15±0.18 $^{P=0.057}$
pT286/tot CaMKII (norm.)	0.95±0.03	1.05±0.11	1.00±0.36	0.95±0.04	0.90±0.22	0.96±0.38	1.06±0.40	0.87±0.13
PLN/GAPDH (norm.)	1.02±0.05	0.98±0.04	1.06±0.04	1.04±0.04	1.04±0.12	1.04±0.04	0.94±0.12	1.02±0.10
pS16/tot PLN (norm.)	0.94±0.05	1.06±0.12	0.95±0.05	0.86±0.04	0.91±0.05	0.93±0.02	1.14±0.14	1.00±0.09
pT17/tot PLN (norm.)	0.93±0.04	1.07±0.06	0.89±0.10	0.87±0.05	0.87±0.02	0.90±0.10	1.15±0.12	0.98±0.05

Table S3. Sex-differences in the expression and phosphorylation of key Ca^{2+} handling proteins in empagliflozin-treated HFpEF mice. Male-female comparisons were made in each experimental group. Expession levels are normalized to WT+Vehicle control without empagliflozin (Empa) treatment. Male-female differences were determined in each group using Mann-Whitney *U*-test. *P* values are reported (if $P \leq 0.10$) after each value in females.

(WT, wild-type; Empa, empagliflozin; Aldo, aldosterone; SERCA2, sarcoplasmic reticulum Ca^{2+} ATPase 2; GAPDH, glyceraldehyde 3-phosphate dehydrogenase; CaMKII, Ca^{2+}/calmodulin-dependent protein kinase II; pT286/tot CaMKII, autophosphorlyation of CaMKII at threonine 286 site to total CaMKII; PLN, phospholamban; pS16/tot PLN, phosphorylation of PLN at serine 16 site to total PLN; pT17/tot PLN, phosphorylation of PLN at threonine 17 site to total PLN)

SUPPLEMENTAL FIGURE AND FIGURE LEGEND

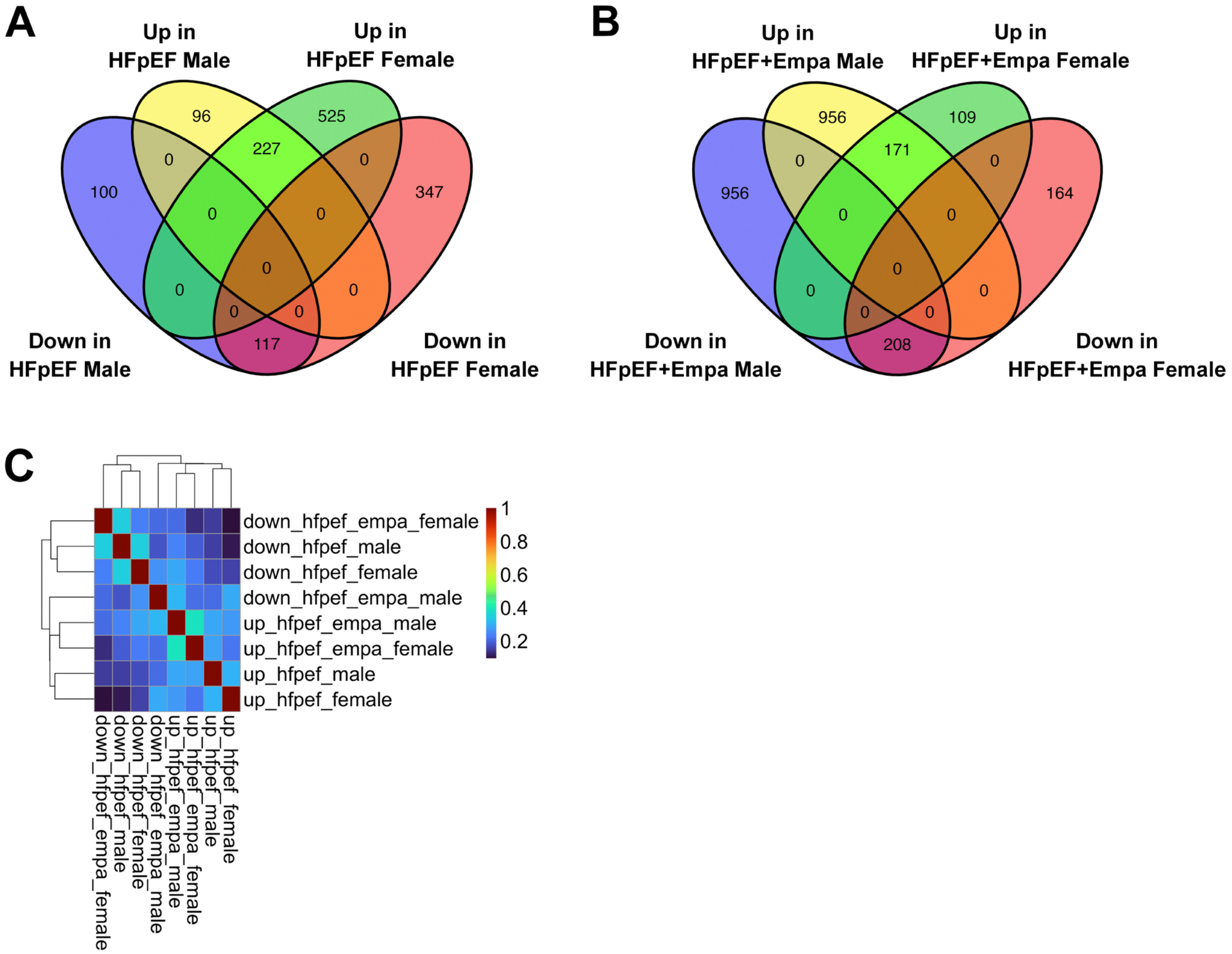

Figure S1. Sex-specific differentially expressed genes in HFpEF with and without empagliflozin treatment.
A, Venn diagram of sex-specific differentially expressed genes (5% false discovery rate threshold, Benjamini-Hochberg correction) in HFpEF hearts from *db/db* mice with chronic aldosterone infusion (*db/db*+Aldo). **B**, Venn diagram of sex-specific differentially expressed genes in the hearts of *db/db*+Aldo mice following 4-wk *in vivo* empagliflozin (Empa) treatment. **C**, Heatmap showing pairwise Jaccard similarity index of shared Gene Ontology/Biological Processes (GO:BP) term enrichment between differentially expressed genes (P<0.05) in each comparison.

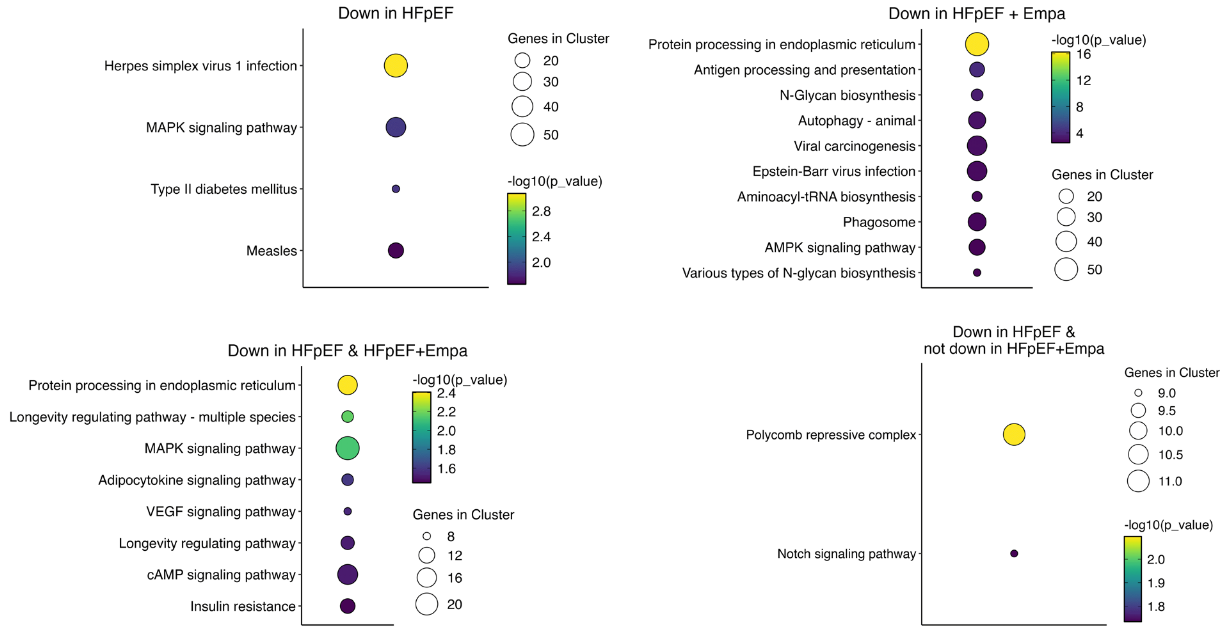

Figure S2. Downregulated Gene Ontology: Biological Processes (GO:BP) and Kyoto Encyclopedia of Genes and Genomes (KEGG) terms in HFpEF with and without empagliflozin treatment.
GO:BP and KEGG pathway term enrichment based on genes downregulated in HFpEF hearts with or without empagliflozin (Empa) treatment. Color of dot represents Benjamini-Hochberg–corrected *P* value (false discovery rate <5%). Size of dot is proportional to the number of differentially expressed genes in that category. If >10 GO:BP and KEGG terms were statistically significant, the top 10 are shown.
(MAPK, mitogen-activated protein kinase; AMPK, AMP-activated protein kinase; VEGF, vascular endothelial growth factor)

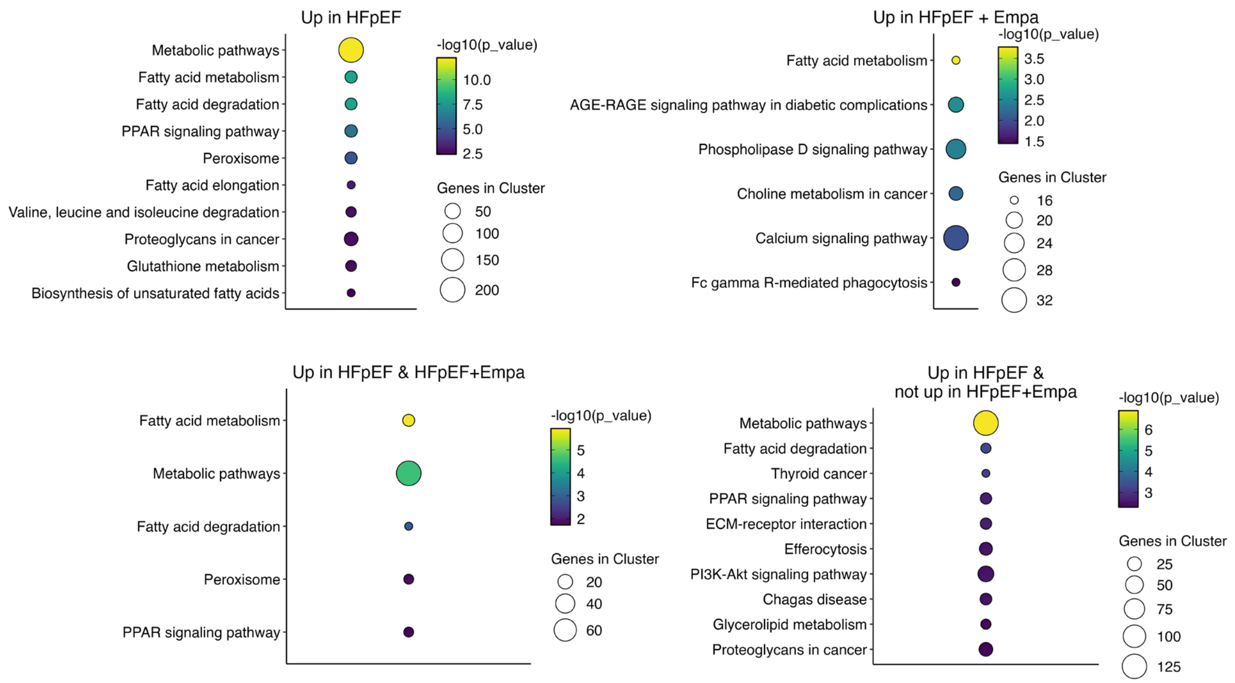

Figure S3. Upregulated Gene Ontology: Biological Processes (GO:BP) and Kyoto Encyclopedia of Genes and Genomes (KEGG) terms in HFpEF with and without empagliflozin treatment.
GO:BP and KEGG pathway term enrichment based on genes upregulated in HFpEF hearts with or without empagliflozin (Empa) treatment. Color of dot represents Benjamini-Hochberg–corrected *P* value (false discovery rate <5%). Size of dot is proportional to the number of differentially expressed genes in that category. If >10 GO:BP and KEGG terms were statistically significant, the top 10 are shown.
(PPAR, peroxisome proliferator-activated receptor; AGE, advanced glycation end-products; RAGE, Receptor for AGE; ECM, extracellular matrix; PI3K, phosphoinositide-3-kinase; Akt, protein kinase B)

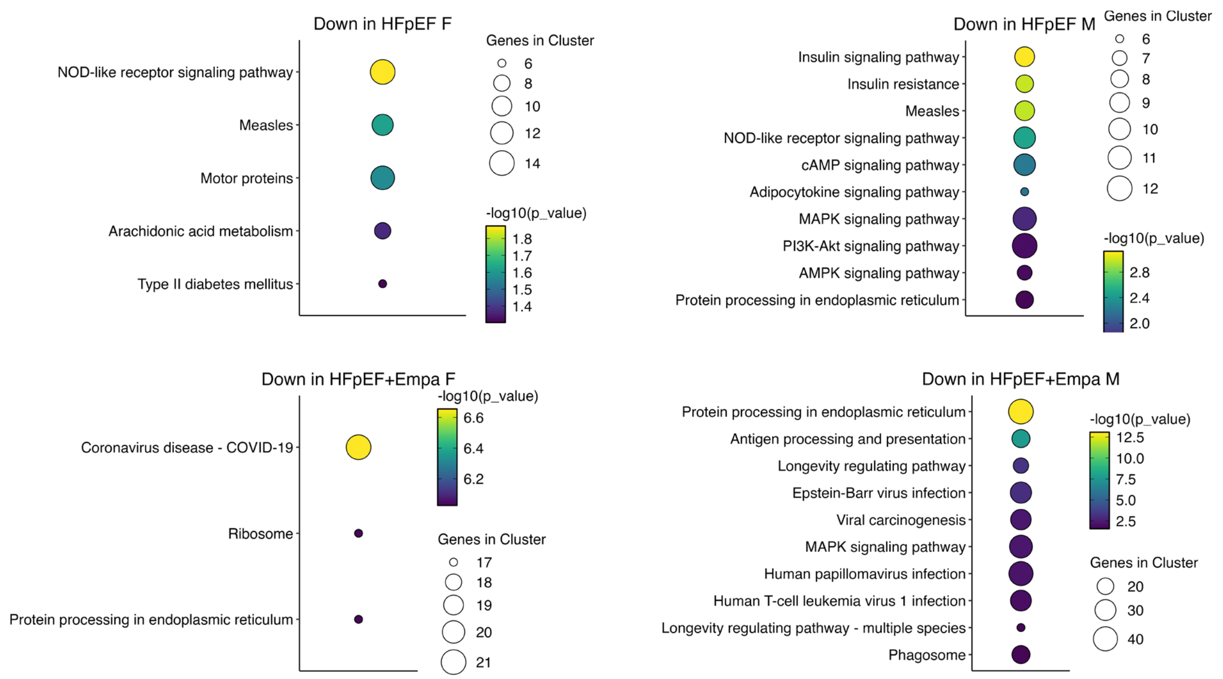

Figure S4. Sex-specific downregulated Gene Ontology: Biological Processes (GO:BP) and Kyoto Encyclopedia of Genes and Genomes (KEGG) term gene clusters in HFpEF with and without empagliflozin treatment.
Sex-specific GO:BP and KEGG pathway term enrichment based on genes downregulated in HFpEF hearts with or without empagliflozin (Empa) treatment. Color of dot represents Benjamini-Hochberg–corrected *P* value (false discovery rate <5%). Size of dot is proportional to the number of differentially expressed genes in that category. If >10 GO:BP and KEGG terms were statistically significant, the top 10 are shown.
(NOD, nucleotide binding oligomerization domain; MAPK, mitogen-activated protein kinase; PI3K, phosphoinositide-3-kinase; Akt, protein kinase B; AMPK, AMP-activated protein kinase)

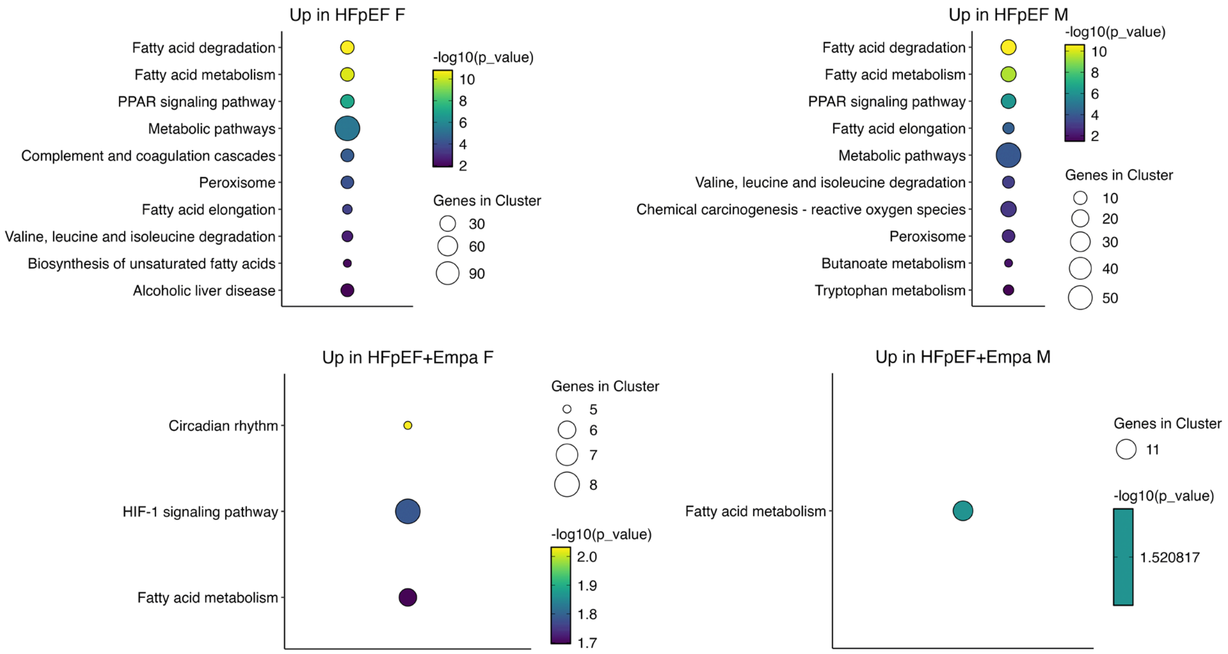

Figure S5. Sex-specific upregulated Gene Ontology: Biological Processes (GO:BP) and Kyoto Encyclopedia of Genes and Genomes (KEGG) term gene clusters in HFpEF with and without empagliflozin treatment.

Sex-specific GO:BP and KEGG pathway term enrichment based on genes upregulated in HFpEF hearts with or without empagliflozin (Empa) treatment. Color of dot represents Benjamini-Hochberg–corrected *P* value (false discovery rate <5%). Size of dot is proportional to the number of differentially expressed genes in that category. If >10 GO:BP and KEGG terms were statistically significant, the top 10 are shown.
(PPAR, peroxisome proliferator-activated receptor; HIF-1, hypoxia-inducible factor 1)

Supporting References

1. Dobin A, Davis CA, Schlesinger F, Drenkow J, Zaleski C, Jha S, Batut P, Chaisson M, Gingeras TR (2013) STAR: ultrafast universal RNA-seq aligner. Bioinformatics 29:15-21 doi:10.1093/bioinformatics/bts635
2. Kolberg L, Raudvere U, Kuzmin I, Vilo J, Peterson H (2020) gprofiler2 -- an R package for gene list functional enrichment analysis and namespace conversion toolset g:Profiler. F1000Res 9 doi:10.12688/f1000research.24956.2
3. Liao Y, Smyth GK, Shi W (2014) featureCounts: an efficient general purpose program for assigning sequence reads to genomic features. Bioinformatics 30:923-930 doi:10.1093/bioinformatics/btt656
4. Love MI, Huber W, Anders S (2014) Moderated estimation of fold change and dispersion for RNA-seq data with DESeq2. Genome Biol 15:550 doi:10.1186/s13059-014-0550-8
5. Ritchie ME, Phipson B, Wu D, Hu Y, Law CW, Shi W, Smyth GK (2015) limma powers differential expression analyses for RNA-sequencing and microarray studies. Nucleic Acids Res 43:e47 doi:10.1093/nar/gkv007
